# Integrative taxonomy of New Caledonian beetles: species delimitation and definition of the *Uloma isoceroides* species group (Coleoptera, Tenebrionidae, Ulomini), with the description of four new species

**DOI:** 10.3897/zookeys.415.6623

**Published:** 2014-06-12

**Authors:** Laurent Soldati, Gael J. Kergoat, Anne-Laure Clamens, Hervé Jourdan, Roula Jabbour-Zahab, Fabien L. Condamine

**Affiliations:** 1 NRA, UMR 1062 CBGP (INRA, IRD, CIRAD, Montpellier SupAgro), Campus de Baillarguet, 34988, Montferrier-sur-Lez, France; 2 IRD, UMR 237 IMBE (IRD, Aix-Marseille Université, CNRS, Université d’Avignon et des pays de Vaucluse), Centre IRD de Nouméa, 98848, Nouméa, Nouvelle-Calédonie; 3 CNRS, UMR 5175 CEFE (CNRS, Université Montpellier 2), 1919 Route de Mende, 34293, Montpellier, France; 4 CNRS, UMR 7641 CMAP (CNRS, École Polytechnique), Route de Saclay, 91128, Palaiseau, France

**Keywords:** Biodiversity hotspot, New Caledonia, New species, Phylogenetics, Taxonomy, Systematics, Tenebrionidae, *Uloma*

## Abstract

New Caledonia is an important biodiversity hotspot with much undocumented biodiversity, especially in many insect groups. Here we used an integrative approach to explore species diversity in the tenebrionid genus *Uloma* (Coleoptera, Tenebrionidae, Ulomini), which encompasses about 150 species, of which 22 are known from New Caledonia. To do so, we focused on a morphologically homogeneous group by comparing museum specimens with material collected during several recent field trips. We also conducted molecular phylogenetic analyses based on a concatenated matrix of four mitochondrial and three nuclear genes for 46 specimens. The morphological study allowed us to discover and describe four new species that belong to the group of interest, the *Uloma isoceroides* group. Molecular analyses confirmed the species boundaries of several of the previously described species and established the validity of the four new species. The phylogenetic analyses also provided additional information on the evolutionary history of the group, highlighting that a species that was thought to be unrelated to the group was in fact a member of the same evolutionary lineage. Molecular species delimitation confirmed the status of the sampled species of the group and also suggested some hidden (cryptic) biodiversity for at least two species of the group. Altogether this integrative taxonomic approach has allowed us to better define the boundaries of the *Uloma isoceroides* species group, which comprises at least 10 species: *Uloma isoceroides* (Fauvel, 1904), *Uloma opacipennis* (Fauvel, 1904), *Uloma caledonica* Kaszab, 1982, *Uloma paniei* Kaszab, 1982, *Uloma monteithi* Kaszab, 1986, *Uloma robusta* Kaszab, 1986, *Uloma clamensae* **sp. n.**, *Uloma condaminei* **sp. n.**, *Uloma jourdani* **sp. n.**, and *Uloma kergoati* **sp. n.** We advocate more studies on other New Caledonian groups, as we expect that much undocumented biodiversity can be unveiled through the use of similar approaches.

## Introduction

New Caledonia, situated in the southwestern part of the Pacific region, is an old oceanic island that is considered as an important biodiversity hotspot (Myers et al. 2000; Lowry et al. 2004). As such it harbours a high concentration of endemic species, especially in evergreen forests that are endangered by nickel mining, human-caused wildfires and biological invasions (Lowry et al. 2004). To counter these threats more surveys are needed, to gain a better knowledge of the species richness and its distribution, which is desperately needed to support the establishment of relevant conservation policies (Bouchet et al. 1995; Mittermeier et al. 1996; Gargominy et al. 1996; Bouchet et al. 1998; Pascal et al. 2008).

Through the advent of molecular systematics, taxonomists have increased species discoveries and documented unsuspected cryptic biodiversity on biodiversity hotspots (Pons et al. 2006; Monaghan et al. 2009; Vieites et al. 2009). For New Caledonia, several phylogenetic studies have been carried out on various endemic groups (e.g. Swenson et al. 2001; Bartish et al. 2005; Murienne et al. 2005; Robillard and Desutter-Grandcolas 2006; Balke et al. 2007a; Smith et al. 2007; Espeland et al. 2008; Murienne et al. 2008; Sharma and Giribet 2009; Espeland and Johanson 2010; Cruaud et al. 2012; Heads 2013). New Caledonian biodiversity is thought to be very ancient and slow accumulating, as attested by local relicts such as tree ferns, conifers (e.g. *Agathis* and *Araucaria*), early angiosperm lineages (e.g. *Amborella*), more derived angiosperms (e.g. *Nothofagus*, palm trees, Proteaceae), unique birds (*Rhynochetos*), or harvestman invertebrates (Troglosironidae). Though the presence of these lineages is often interpreted as an indication of old vicariance events (Ladiges and Cantrill 2007; Heads 2008, 2013) numerous studies have indicated that the contribution of recent dispersals events is more likely (see Grandcolas et al. 2008; Keppel et al. 2009; Espeland and Murienne 2011; Cruaud et al. 2012; Pillon 2012 for reviews or meta-analyses). The geological evidence also emphasizes a dynamic recent history (Cluzel et al. 2001; Pelletier 2006; Schellart et al. 2006 but see Ladiges and Cantrill 2007; Heads 2013). The fact that most clades appear to have recently diversified implies that the morphological differentiation between species may be shallow and hard to detect even for specialists, which argues in favour of more integrative taxonomic approaches mixing molecular, morphological, ecological, and geographic data (Padial et al. 2010; Schlick-Steiner et al. 2010).

Because New Caledonia is still subjected to numerous threats (biological invasions, mining, forest logging and burning), a particular effort must be undergone to discover, document and protect its unique biodiversity. Although its categorization as a biodiversity hotspot is based on estimates of diversity on vascular plants and vertebrate groups, it likely also applies to other groups such as insects (Stork and Habel 2014). The insect fauna of New Caledonia included about 4000 known species in 1993 with an estimated total of 16,000 (Chazeau 1993). Specific surveys of various groups of New Caledonian insects have underlined very high proportions of endemics species (e.g. Balke et al. 2007b; Kuschel 2008; Espeland and Johanson 2010), which parallel those of plants (Novotny et al. 2006). Other factors such as environmental filtering (e.g. role of ultramafic soils; Ladiges and Cantrill 2007; Espeland et al. 2008; Pillon et al. 2010) may also be invoked to explain this pattern. An example of recent increase in taxonomic knowledge through both morphology and molecular studies is in caddisflies (Trichoptera), for which 132 species were initially recorded from New Caledonia (of which 130 are endemic) (see also Balke et al. 2007b). Fifty-eight more species were recently discovered using a combination of data (Malm and Johanson 2007; Espeland and Johanson 2008a, b; Johanson and Keijsner 2008; Malm and Johanson 2008a, b; Oláh and Johanson 2008), and more than 200 undescribed species so far are present in the collections at the Swedish Museum of Natural History (Espeland et al. 2008). Altogether this demonstrates the need for a more complete biodiversity inventory in order to set more adequate conservation priorities for the future.

In the darkling beetle family (Coleoptera, Tenebrionidae), the proportion of New Caledonian species that are endemic is extremely high (215 out of 234 species; Kaszab 1982, 1986). The species richness of New Caledonian tenebrionids is also likely underestimated, because few studies (and no molecular-based studies) have been conducted on this group since Kaszab’s monographic works on the archipelago (Kaszab 1982, 1986). In this study we chose to focus on *Uloma* (Tenebrioninae, Ulomini), a genus with a worldwide distribution that encompasses at least 150 species (Matsumoto and Nishikawa 1986), of which 22 are endemic to New Caledonia (Kaszab 1982, 1986). Most of these species cannot be reliably assigned to a homogeneous species group (Kaszab 1982, 1986). The only exception is a group of five species (*Uloma caledonica*, *Uloma isoceroides*, *Uloma monteithi*, *Uloma paniei* and *Uloma robusta*), which share the following combination of characters: (i) head short and broad; (ii) male with clypeus and frons located in the same plane, not impressed along the frontoclypeal suture, flat, with a shagreened dull surface; (iii) metathorax very short; (iv) flightless.

Here we aim at exploring species diversity in this group by comparing the specimens we collected through several field missions in New Caledonia with material from several collections and museums. We also use molecular phylogenetics that allows us to: (i) reconstruct the evolutionary history of the group; (ii) assess species boundaries within the group and confirm the existence of potential new species.

## Material and methods

### Sampling of specimens

Specimens were collected during several biodiversity surveys undergone between March 2008 and November 2011 in New Caledonia (project ANR BIONEOCAL). Most specimens were caught by hand through a careful examination of fallen branches, rotten logs and standing trees (either unhealthy or dead). In addition, we used headlamps at night to find and collect specimens where they were most active. For this study we tentatively included all specimens that possibly belonged to the group of interest. We also included specimens from *Uloma opacipennis*, as preliminary analyses conducted on a larger molecular dataset indicate that this species is potentially a member of the group of interest. Morphological examinations of specimens allowed us to determine that the sampled specimens likely corresponded to seven distinct morphospecies (see Table 1 and the Taxonomy results), of which four could not be assigned to any known species. As outgroups, we also used two morphologically unrelated species of *Uloma* that are not distributed in New Caledonia (*Uloma freyi* endemic to the Fiji Islands, and *Uloma rufa* widespread in Europe). *Uloma rufa* was used to root the tree based on the results of Kergoat et al. (2014).

**Table 1.** Taxon sampling. All specimens are from New Caledonia with the exception of the individuals of *Uloma freyi* and *Uloma rufa*.

Systematics			GenBank accession No.						
Species	Voucher No.	Locality	12S	16S	Cyt b	COI	28SD2-D3	Wingless	18S
**Family Tenebrionidae Latreille, 1802**									
Subfamily Tenebrioninae Latreille, 1802									
Tribe Ulomini Blanchard, 1845									
*Uloma caledonica* Kaszab, 1982	**LSOL.01828**	‘Parc de la Rivière bleue’	KJ510053	-missing-	-missing-	-missing-	-missing-	-missing-	-missing-
*Uloma caledonica* Kaszab, 1982	**LSOL.02085**	‘Parc de la Rivière bleue’	KJ510054	-missing-	-missing-	-missing-	-missing-	-missing-	-missing-
*Uloma clamensae* sp. n.	**LSOL.01336**	‘Putchaté, Atéu’	KJ510055	KJ510095	KJ510021	KJ509982	KJ510159	KJ510042	KJ510127
*Uloma clamensae* sp. n.	**LSOL.02021**	‘Massif des Lèvres’	KJ510056	KJ510096	-missing-	KJ509983	KJ510160	-missing-	KJ510128
*Uloma condaminei* sp. n.	**LSOL.02108**	‘Wayem, Panié’	-missing-	-missing-	-missing-	KJ509984	-missing-	-missing-	-missing-
*Uloma condaminei* sp. n.	**LSOL.02126**	‘Wayem, Panié’	KJ510057	-missing-	-missing-	KJ509985	-missing-	-missing-	-missing-
*Uloma condaminei* sp. n.	**LSOL.02127**	‘Wayem, Panié’	KJ510058	KJ510097	-missing-	KJ509986	-missing-	-missing-	-missing-
*Uloma condaminei* sp. n.	**LSOL.02129**	‘Wayem, Panié’	KJ510059	KJ510098	-missing-	KJ509987	-missing-	-missing-	-missing-
*Uloma condaminei* sp. n.	**LSOL.02130**	‘Wayem, Panié’	KJ510060	KJ510099	-missing-	KJ509988	KJ510161	-missing-	KJ510129
*Uloma condaminei* sp. n.	**LSOL.02131**	‘Wayem, Panié’	-missing-	KJ510100	-missing-	-missing-	-missing-	KJ510043	-missing-
*Uloma condaminei* sp. n.	**LSOL.02142**	‘Wayem, Panié’	KJ510061	-missing-	-missing-	KJ509989	-missing-	-missing-	-missing-
*Uloma condaminei* sp. n.	**LSOL.02147**	‘Wayem, Panié’	KJ510062	KJ510101	-missing-	KJ509990	KJ510162	-missing-	KJ510130
*Uloma freyi* Kulzer, 1960	**LSOL.00996**	(Fiji islands)	KJ510063	KJ510102	KJ510022	KJ509991	KJ510163	KJ510044	KJ510131
*Uloma isoceroides* (Fauvel, 1904)	**LSOL.01144**	‘Plateau de Dogny’	KJ510064	KJ510103	-missing-	KJ509992	KJ510164	-missing-	KJ510132
*Uloma isoceroides* (Fauvel, 1904)	**LSOL.01250**	‘Massif de la Tchamba’	KJ510065	KJ510104	KJ510023	KJ509993	KJ510165	-missing-	KJ510133
*Uloma jourdani* sp. n.	**LSOL.02158**	‘Wewec, Panié’	KJ510066	KJ510105	KJ510024	KJ509994	-missing-	-missing-	KJ510134
*Uloma jourdani* sp. n.	**LSOL.02209**	‘La Guen, Panié’	KJ510067	-missing-	-missing-	KJ509995	-missing-	-missing-	-missing-
*Uloma jourdani* sp. n.	**LSOL.02242**	‘La Guen, Panié’	KJ510068	KJ510106	KJ510025	KJ509996	KJ510166	-missing-	KJ510135
*Uloma jourdani* sp. n.	**LSOL.02243**	‘La Guen, Panié’	KJ510069	KJ510107	KJ510026	KJ509997	-missing-	-missing-	KJ510136
*Uloma jourdani* sp. n.	**LSOL.02201**	‘Dawenia, Panié’	KJ510070	KJ510108	-missing-	KJ509998	KJ510167	-missing-	KJ510137
*Uloma jourdani* sp. n.	**LSOL.02202**	‘Dawenia, Panié’	KJ510071	KJ510109	-missing-	KJ509999	KJ510168	-missing-	KJ510138
*Uloma jourdani* sp. n.	**LSOL.02263**	‘Dawenia, Panié’	KJ510072	KJ510110	KJ510027	KJ510000	-missing-	KJ510045	KJ510139
*Uloma jourdani* sp. n.	**LSOL.02265**	‘Dawenia, Panié’	KJ510073	KJ510111	-missing-	KJ509101	-missing-	-missing-	KJ510140
*Uloma jourdani* sp. n.	**LSOL.02292**	‘Dawenia, Panié’	KJ510074	KJ510112	-missing-	KJ509102	KJ510169	KJ510046	KJ510141
*Uloma jourdani* sp. n.	**LSOL.02294**	‘Dawenia, Panié’	KJ510075	KJ510113	KJ510028	KJ509103	-missing-	KJ510047	KJ510142
*Uloma kergoati* sp. n.	**LSOL.01012**	‘Monts Koghis’	KJ510076	-missing-	-missing-	KJ509104	-missing-	-missing-	KJ510143
*Uloma kergoati* sp. n.	**LSOL.01122**	‘Monts Koghis’	KJ510077	-missing-	-missing-	-missing-	-missing-	-missing-	KJ510144
*Uloma kergoati* sp. n.	**LSOL.01587**	‘Monts Koghis’	KJ510078	KJ510114	KJ510029	KJ509105	KJ510170	-missing-	KJ510145
*Uloma kergoati* sp. n.	**LSOL.01805**	‘Monts Koghis’	KJ510079	-missing-	KJ510030	KJ509106	-missing-	-missing-	KJ510146
*Uloma kergoati* sp. n.	**LSOL.01806**	‘Monts Koghis’	KJ510080	-missing-	KJ510031	KJ509107	-missing-	-missing-	KJ510147
*Uloma opacipennis* (Fauvel, 1904)	**LSOL.01020**	‘Mont Do’	KJ510081	-missing-	-missing-	KJ509108	-missing-	-missing-	KJ510148
*Uloma opacipennis* (Fauvel, 1904)	**LSOL.01360**	‘Parc de la Rivière bleue’	KJ510082	KJ510115	KJ510032	-missing-	-missing-	-missing-	KJ510149
*Uloma opacipennis* (Fauvel, 1904)	**LSOL.02144**	‘Wayem, Panié’	KJ510083	-missing-	-missing-	KJ510009	KJ510171	-missing-	-missing-
*Uloma opacipennis* (Fauvel, 1904)	**LSOL.02184**	‘Dawenia, Panié’	KJ510084	KJ510116	KJ510033	KJ510010	KJ510172	KJ510048	-missing-
*Uloma opacipennis* (Fauvel, 1904)	**LSOL.02185**	‘Dawenia, Panié’	KJ510085	KJ510117	KJ510034	KJ510011	KJ510173	-missing-	-missing-
*Uloma opacipennis* (Fauvel, 1904)	**LSOL.02193**	‘Dawenia, Panié’	KJ510086	KJ510118	KJ510035	KJ510012	KJ510174	KJ510049	KJ510150
*Uloma opacipennis* (Fauvel, 1904)	**LSOL.02206**	‘Dawenia, Panié’	KJ510087	-missing-	-missing-	KJ510013	-missing-	-missing-	-missing-
*Uloma opacipennis* (Fauvel, 1904)	**LSOL.02224**	‘La Guen, Panié’	KJ510088	KJ510119	KJ510036	-missing-	-missing-	-missing-	KJ510151
*Uloma opacipennis* (Fauvel, 1904)	**LSOL.02225**	‘La Guen, Panié’	KJ510089	KJ510120	KJ510037	KJ510014	-missing-	-missing-	KJ510152
*Uloma opacipennis* (Fauvel, 1904)	**LSOL.02236**	‘La Guen, Panié’	KJ510090	KJ510121	-missing-	KJ510015	-missing-	KJ510050	KJ510153
*Uloma opacipennis* (Fauvel, 1904)	**LSOL.02237**	‘La Guen, Panié’	KJ510091	KJ510122	-missing-	KJ510016	-missing-	KJ510051	KJ510154
*Uloma opacipennis* (Fauvel, 1904)	**LSOL.02250**	‘La Guen, Panié’	KJ510092	KJ510123	KJ510038	KJ510017	KJ510175	KJ510052	KJ510155
*Uloma opacipennis* (Fauvel, 1904)	**LSOL.02251**	‘La Guen, Panié’	KJ510093	KJ510124	KJ510039	KJ510018	-missing-	-missing-	KJ510156
*Uloma opacipennis* (Fauvel, 1904)	**LSOL.02260**	‘Dawenia, Panié’	-missing-	KJ510125	KJ510040	KJ510019	-missing-	-missing-	KJ510157
*Uloma opacipennis* (Fauvel, 1904)	**LSOL.02261**	‘Dawenia, Panié’	KJ510094	KJ510126	KJ510041	KJ510020	-missing-	-missing-	KJ510158
*Uloma rufa* (Piller & Mitterbacher, 1783)	**U.rufa.1**	(France)	KC160347	KC160424	-missing-	-missing-	-missing-	-missing-	KJ003714

### DNA extraction and sequencing

Total DNA of 46 specimens was extracted following the non-invasive protocol of extraction of Gilbert et al. (2007). Four mitochondrial gene fragments were sequenced, namely 687 bp of the cytochrome oxidase I (COI), 458 bp of the cytochrome b (Cyt b), 380 bp of the ribosomal 12S RNA (12S), and 532 bp of the ribosomal 16S RNA (16S). Three nuclear gene regions were sequenced, namely 746 bp of the domain D2-D3 of the 28S ribosomal DNA (28SD2-D3), 459 bp of wingless (Wg), and 1881 bp of the 18S ribosomal DNA (18S). All these genes were chosen because they are known to be informative in phylogenetic analyses of tenebrionid beetles (Papadopoulou et al. 2009, 2010; Condamine et al. 2013) or in other coleopteran groups (McKenna et al. 2009; Kergoat et al. 2011; Deuve et al. 2012). Polymerase chain reaction amplifications were performed with standard settings for primer sequences and thermocycler procedures (see Belshaw and Quicke 2002; Kergoat et al. 2004, 2005; Wild and Maddison 2008 for additional information).

The PCR products were processed by the French sequencing centre Genoscope using a BigDye 3.1 sequencing kit and Applied 3730xl sequencers. The resulting sequences of complementary strands were further edited and reconciled using Geneious 5.1 (available at: www.geneious.com). All the sequences generated in this study were deposited in GenBank (KJ509982-KJ51017, see Table 1 for details). For all protein-coding genes (COI, Cyt b and Wg), we used Mesquite 2.75 (available at: www.mesquiteproject.org) to check coding frames for possible errors or stop codons. Alignment of non-coding genes (12S, 16S, 28SD2-D3, and 18S) was carried out using Muscle (Edgar 2004) with default option settings. The combination of the seven gene fragments resulted in a matrix of 46 taxa and 5143 aligned characters.

### Phylogenetic analyses

Maximum likelihood (ML) analyses were performed with the raxmlGUI package v1.3 (Silvestro and Michalak 2012), which relies on RAxML v.7.4.2 executables (Stamatakis 2006). We used partitioned analyses (Nylander et al. 2004) with one partition for the mitochondrial genes and one partition for the nuclear genes. For each partition, we combined a general time reversible (GTR) substitution model with a CAT (category) model, which optimizes the evolutionary rate of individual sites using a fixed number of rate categories. To account for by the fact that numerous sites were invariable we also added an additional parameter to the model (+I; proportion of invariable sites). Then we conducted 100 independent runs with corresponding GTR+CAT+I models. Support of trees was assessed using 1000 non-parametric bootstrap replicates. Nodes supported by bootstrap values (BV) ≥ 70% were considered as strongly supported following Hillis and Bull (1993).

To determine putative molecular species clusters on our dataset we then use Poisson tree processes (PTP) models (Zhang et al. 2013). Because this approach does not require ultrametrization of trees (and its associated biases), it constitutes an elegant alternative to other species delineation models such as the General mixed Yule coalescent model of Pons et al. (2006). With the PTP model, speciation or branching events are modelled in terms of number of substitutions (represented by branch lengths), so it only requires a phylogenetic input tree. Corresponding analyses were conducted on the web server for PTP (available at http://species.h-its.org/ptp/) using the best ML tree resulting from the raxmlGUI analysis.

### Morphological study

Specimens examined for this study are deposited in the following institutions and collections (all collection codes follow Evenhuis (2008)):

BMNH The Natural History Museum, London, United Kingdom.

BPBM Hawaii, Bernice P. Bishop Museum, Honolulu, USA.

CBGP Centre de Biologie pour la Gestion des Populations, Montferrier-sur-Lez, France.

CS Collection Soldati, Montpellier, France.

Hnhm Hungarian Natural History Museum, Budapest, Hungary.

IRSNB Institut Royal des Sciences Naturelles de Belgique, Brussels, Belgium.

MNHN Muséum National d’Histoire Naturelle, Paris, France.

MTD Museum für Tierkunde, Dresden, Germany.

QM Queensland Museum, Brisbane, Australia.

USNM National Museum of Natural History, Washington D.C., USA.

Specimens were glued on glue boards, then pinned, labelled and dry stored in insect boxes. The glue used (Cléopâtre™ ref. AD110P) to secure the specimens on the glue boards is water soluble and completely reversible. Male genitalia were also dissected and glued on the same glue board that their respective specimens. Pictures of specimens were taken by L. Soldati using the focus stacking system Entovision™ on the imaging platform of the CBGP. Morphological terms used in this study follow the terminology of Matthews and Bouchard (2008) and Matthews et al. (2010).

## Results

### Molecular phylogenetics

**Figure 1.**
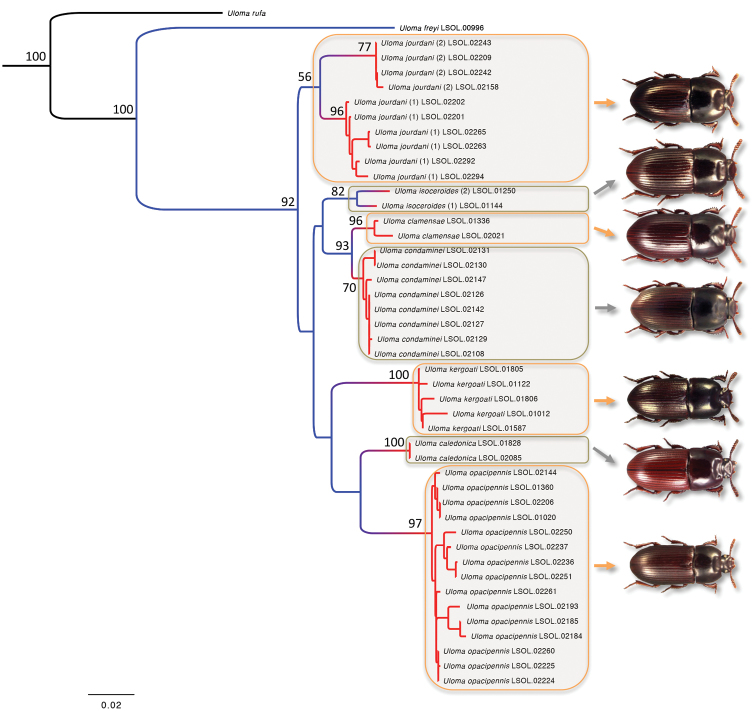
Maximum likelihood tree resulting from the analysis of the combined dataset. Support of major nodes is provided by BV (only BV ≥ 50% are figured). For the group of interest we used coloured frames to highlight the seven sampled morphospecies (*Uloma caledonica*, *Uloma clamensae*, *Uloma condaminei*, *Uloma isoceroides*, *Uloma jourdani*, *Uloma kergoati* and *Uloma opacipennis*). On the right, corresponding male habitus are also included for illustrative purpose. Results of the PTP analysis are provided using coloured branches. Putative molecular species are indicated using transitions between blue-coloured branches to red-coloured branches. For the two cases (for *Uloma isoceroides* and *Uloma jourdani*) in which two distinct putative species clusters are inferred we added numbers into brackets to indicate the assignation of specimens to a specific species cluster.

The ML analyses yield a best ML tree with a likelihood score of -11607.44 (Fig. 1). All the nodes that lead to putative taxa (i.e. morphospecies) are well-supported (BV ≥ 70%). All members of the group of interest are recovered in a well-supported clade (BV of 92%). Within this clade, the representatives of the *Uloma jourdani* sp. n. are in a sister position to all remaining NC representatives. Then, two major clades can be distinguished, each of them corresponding to three morphospecies. In the first, the two representatives of *Uloma isoceroides* are sisters to *Uloma clamensae* sp. n. and *Uloma condaminei* sp. n. In the second *Uloma kergoati* sp. n. is sister to a clade encompassing representatives of *Uloma caledonica* and *Uloma opacipennis*. At the intraspecific level it is also worth highlighting the fact that representatives of *Uloma jourdani* sp. n. are clustered into two well-differentiated clades (respectively supported by a BV of 77% and 96%). Regarding molecular species delimitation, the PTP analyses recover nine putative species clusters (see Fig. 1) for the seven sampled morphospecies belonging to the group of interest. Additional species clusters were found in *Uloma isoceroides* (two distinct clusters encompassing one individual each) and *Uloma jourdani* sp. n. (two distinct clusters encompassing six and four specimens, respectively).

## Taxonomy

The *Uloma isoceroides* species group is named after *Uloma isoceroides*, the first described species of the group (page 182 in Fauvel 1904). This constitutes 10 species, four of which are new. All but one (*Uloma opacipennis*) can be characterized by the following combination of characters: (i) head short and broad; (ii) male with clypeus and frons located in the same plane, not impressed along the frontoclypeal suture, flat, with a shagreened dull surface; (iii) metathorax very short. Though *Uloma opacipennis* is morphologically distinct from the other members of the group (see the corresponding diagnosis section) its inclusion is fully supported by the results of the molecular analyses.

### Uloma caledonica

Kaszab, 1982

http://species-id.net/wiki/Uloma_caledonica

Figs 2A , 3A–B

Uloma caledonica Kaszab, Folia Entomologica Hungarica 18: 87.

#### Type locality.

Saint Louis, Forêt de Thi.

#### Type specimens.

Holotype male (BPBM). Paratypes: 11 males and 10 females (BPBM), two males and one female (USNM), three males (IRSNB), none examined; one male, original label: “Nouvelle-Calédonie, 1893, Coll. Ed. Fleutiaux” (MNHN); one male, original label: “Nouvelle-Calédonie” (Hnhm), both examined.

#### Diagnosis.

*Uloma caledonica* is one of the four species of the group in which the mentum of the male is completely glabrous and flat. It differs from these three species (*Uloma jourdani*, *Uloma isoceroides* and *Uloma kergoati*) by the longer metaventrite (between meso- and metacoxae approximately as long as a mesocoxa), the humeri slightly developed, the elytral striae of punctures strongly marked and developed to apex, and the pronotal punctation barely visible. The shape of the aedeagus is also unique among the New Caledonian *Uloma* species, with the parameres bottleneck-shaped and triangularly notched at the apex.

**Figure 2.**
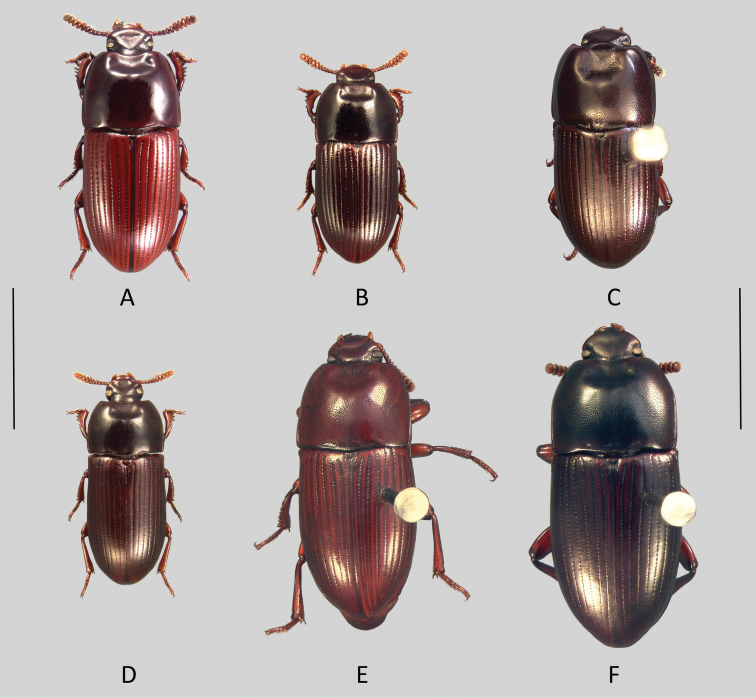
Habitus (dorsal view): **A** *Uloma caledonica* **B** *Uloma isoceroides* **C** *Uloma monteithi* **D** *Uloma opacipennis* **E** *Uloma paniei* **F** *Uloma robusta*. Scale bar: 5 mm.

#### Distribution.

Kaszab (1982: 87) cited this species from the following localities:

Saint-Louis (Forêt de Thi), Rivière Bleue (Yaté), La Couèle-Yaté Rd., Mt Koghi, Nouméa, Île des Pins. “Neukaledonien (Grande Terre SO, Île des Pins)”.

#### Additional localities.

Mont Do (21°45.63'S, 166°00.15'E, ca 940 m) 6.III.2008, L. Soldati, G.J. Kergoat & H. Jourdan rec. (CBGP); Parc Provincial de la Rivière Bleue, Refuge des Ornithologues (22°08.04'E, 166°39.19'S, ca 190 m) 4.XI.2008, L. Soldati, G.J. Kergoat, F.L. Condamine & H. Jourdan rec. (CBGP).

### Uloma clamensae

L. Soldati sp. n.

http://zoobank.org/D693C69B-FC2C-43D0-9BDC-93D7D95D26F5

http://species-id.net/wiki/Uloma_clamensae

**Figure 3.**
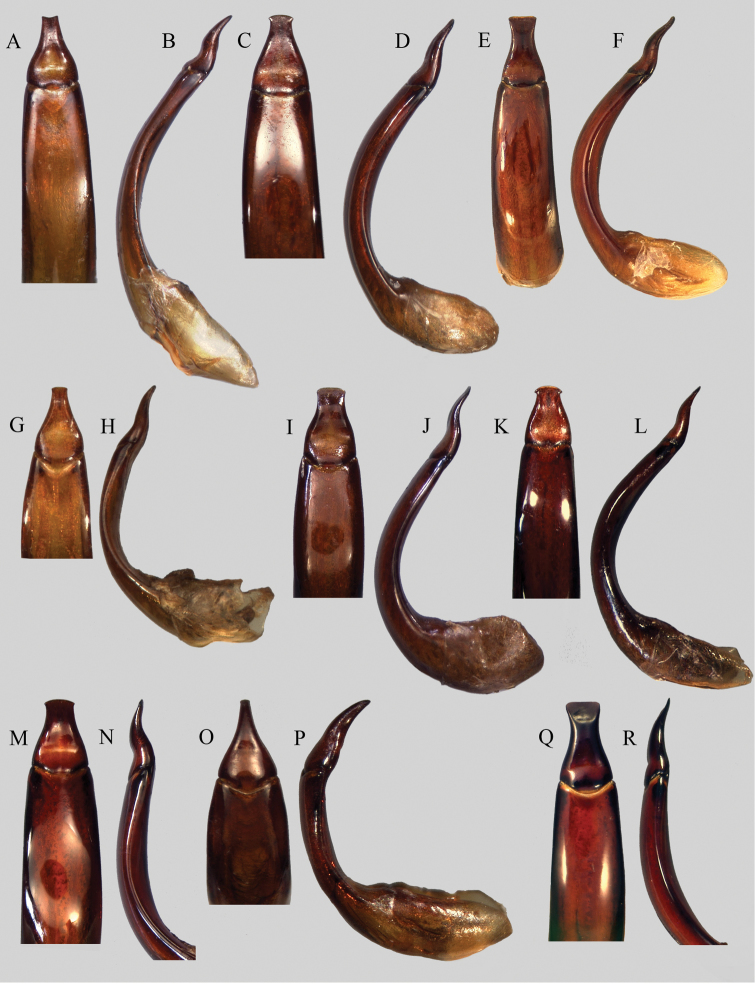
Aedeagus (tergal face and lateral view): **A–B** *Uloma caledonica* **C–D** *Uloma clamensae* **E–F** *Uloma condaminei* **G–H** *Uloma isoceroides* **I–J** *Uloma jourdani* **K–L** *Uloma kergoati* **M–N** *Uloma monteithi* **O–P** *Uloma opacipennis* **Q–R** *Uloma robusta*.

**Figure 4.**
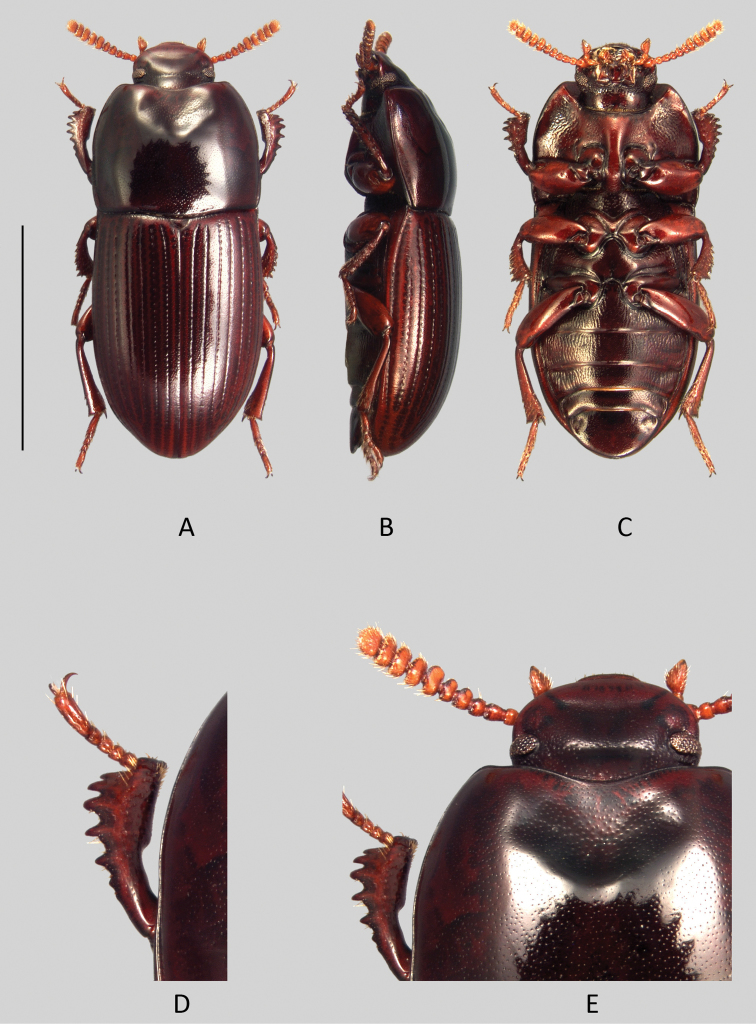
*Uloma clamensae*: **A** habitus (dorsal view) **B** habitus (lateral view) **C** habitus (ventral view) **D** anterior tibia (upper face) **E** head (dorsal view). Scale bar: 5 mm.

Figs 3C–D , 4A, B, C, D, E

#### Type specimens.

Holotype male, pinned, with genitalia glued on the same card as the specimen itself. Original label: “Nouvelle-Calédonie, Putchaté, Atéu, 23.IV.2009, E. Baby leg. / 20°59.39'S, 164°54.04'E, ca 370 m alt.” / *Uloma clamensae* m. n. sp. L. Soldati det. 2013, HOLOTYPE ♂ (red printed label) (MNHN); Paratypes, same data as Holotype: one female (MNHN), one male (CS).

#### Diagnosis.

*Uloma clamensae* is closely related to *Uloma condaminei* sp. n. The two species are so similar that the only reliable way to separate them is to compare their male genitalia. *Uloma clamensae* and *Uloma condaminei* can also be distinguished from all the other *Uloma* species of New Caledonia by the unique structure of the mentum in the male: the mentum pilosity is reduced to two apical hair tufts on each side (Fig. 6F–G).

In the case of isolated females, the geographic distribution may distinguish *Uloma clamensae* from *Uloma condaminei*.

#### Description.

Length 9.0–9.5 mm; width 3.2–3.5 mm. Shining, pitchy dark brown. Antennae, mouthparts, legs and elytra reddish-brown.

Head (Fig. 3E).

Male: Transverse, genae straight in front of the eyes, then continuous in curved line with the clypeus. Frontoclypeal suture superficially impressed. Frons and clypeus fused, with shagreened dull surface, covered with extremely fine, sparse and barely visible punctures. Vertex convex, shining and separated from the frons by a transverse depression that extends behind the eyes. Tempora (densely) and vertex (sparsely) coarsely punctured.

Female: in contrast to male, frontoclypeal area finely and quite densely punctate over a shining background. Frontoclypeal suture shallowly impressed.

Antennae (Fig. 4E) gradually becoming transverse and expanded from antennomere 5. Antennomeres 5–9 flattened with apices more or less protruding in middle, especially 7^th^.

Mentum of the male (Fig. 4C) cordate, with two oblique lateral grooves near the base and two apical dense hair tufts, all arranged symmetrically in relation to midline; disc slightly concave longitudinally, unpunctured and shining. Male mentum of *Uloma clamensae* is similar to the one of *Uloma condaminei* (see Fig. 6F–G). Female mentum cordate but narrower, not transverse, with the two oblique lateral grooves merging at base to form a U-shape in between, disc flat, smooth and shining, without punctation.

Pronotum: about 1.2 times wider than long, sides subparallels, widest around the middle. Rim on the anterior margin at middle usually obliterated, sometimes slightly visible; base unmargined, with exception of two very short folds located at the level of the two concave curves of external margin. Anterior angles 90°but smooth at the top and slightly protruding forward, posterior angles obtuse. Lateral rims becoming progressively thinner from the base toward the anterior angles. Whole upper surface of the pronotum very finely punctate, sparser on the disc but denser on the sides.

Male: antero-median depression of pronotum well impressed, not reaching half of pronotal length, its posterior edge arcuate and delimited by four very faint elevations. The lateral bumps anterolaterally bordering the depression low and not projecting to anterior edge. Interior of depression somewhat more strongly punctate than rest of pronotal surface.

Female: pronotum regularly convex, without antero-median depression and overall finely punctate.

Prosternal process in lateral view obliquely bent beneath procoxae.

Elytra quite convex, humeral angles of lateral margin protruding. Lateral margin barely visible in dorsal view except around middle. Each elytron bears nine grooved and punctured striae and a faint scutellary striole. Strial punctures slightly wider than grooves. Elytral intervals nearly flat on disc and becoming slightly convex laterally and toward apex, covered with fine and superficial punctation.

Metaventrite short, length between meso- and metacoxae less than half the length of mesocoxa.

Abdomen. Abdominal ventrites 1-4 (Fig. 4C) finely and superficially punctate on a narrow median longitudinal strip. On each side of this longitudinal strip, punctation becomes progressively larger and sparser toward the sides and the integument’s surface is slightly striate longitudinally. The apical ventrite covered with fine scattered punctation, its outer margin without rim.

Legs. Anterior tibiae (Fig. 4D) without carina on their upper face and strongly notched at the base of nearly half the length of inner side.

Aedeagus: tergal face (Fig. 3C), with basal two-thirds of parameres bottleneck-shaped, then abruptly enlarged and securiform at the apex. In lateral view (Fig. 3D), parameres bisinuate and narrowed toward apex.

#### Etymology.

This new species is named after A.-L. Clamens, biologist and member of the “All Blaps” team.

#### Distribution.

*Uloma clamensae* is currently only known only from its type locality in New Caledonia.

### Uloma condaminei

L. Soldati sp. n.

http://zoobank.org/8EEBB1B0-79AD-4FEB-930F-FAF3C358805C

http://species-id.net/wiki/Uloma_condaminei

**Figure 5.**
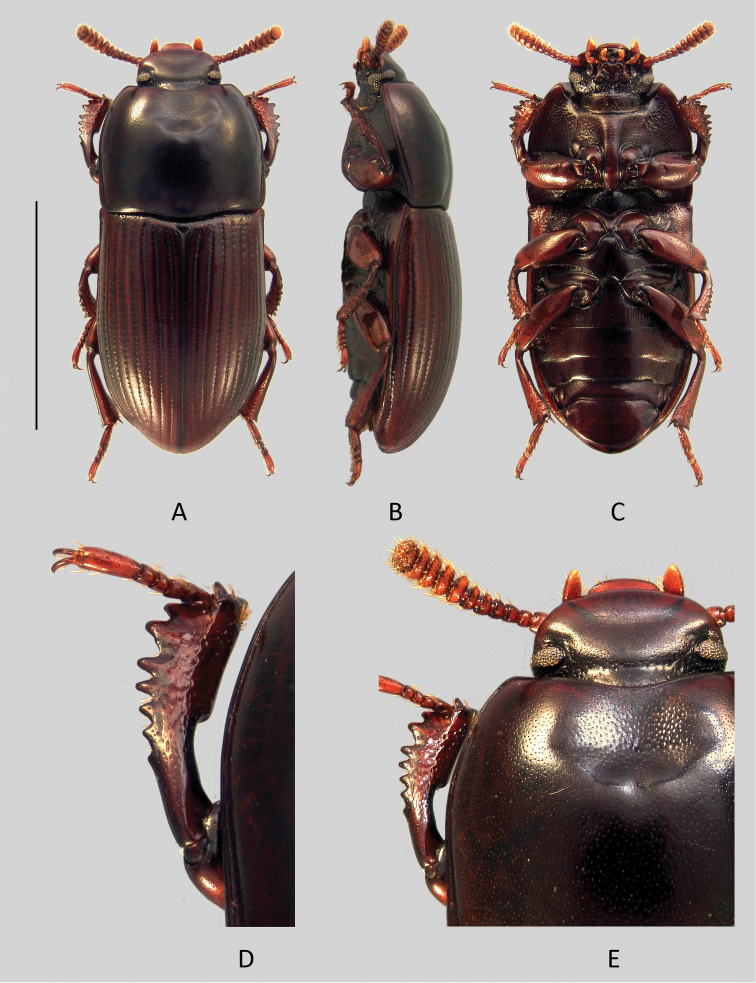
*Uloma condaminei*: **A** habitus (dorsal view) **B** habitus (lateral view) **C** habitus (ventral view) **D** anterior tibia (upper face) **E** head (dorsal view). Scale bar: 5 mm.

**Figure 6.**
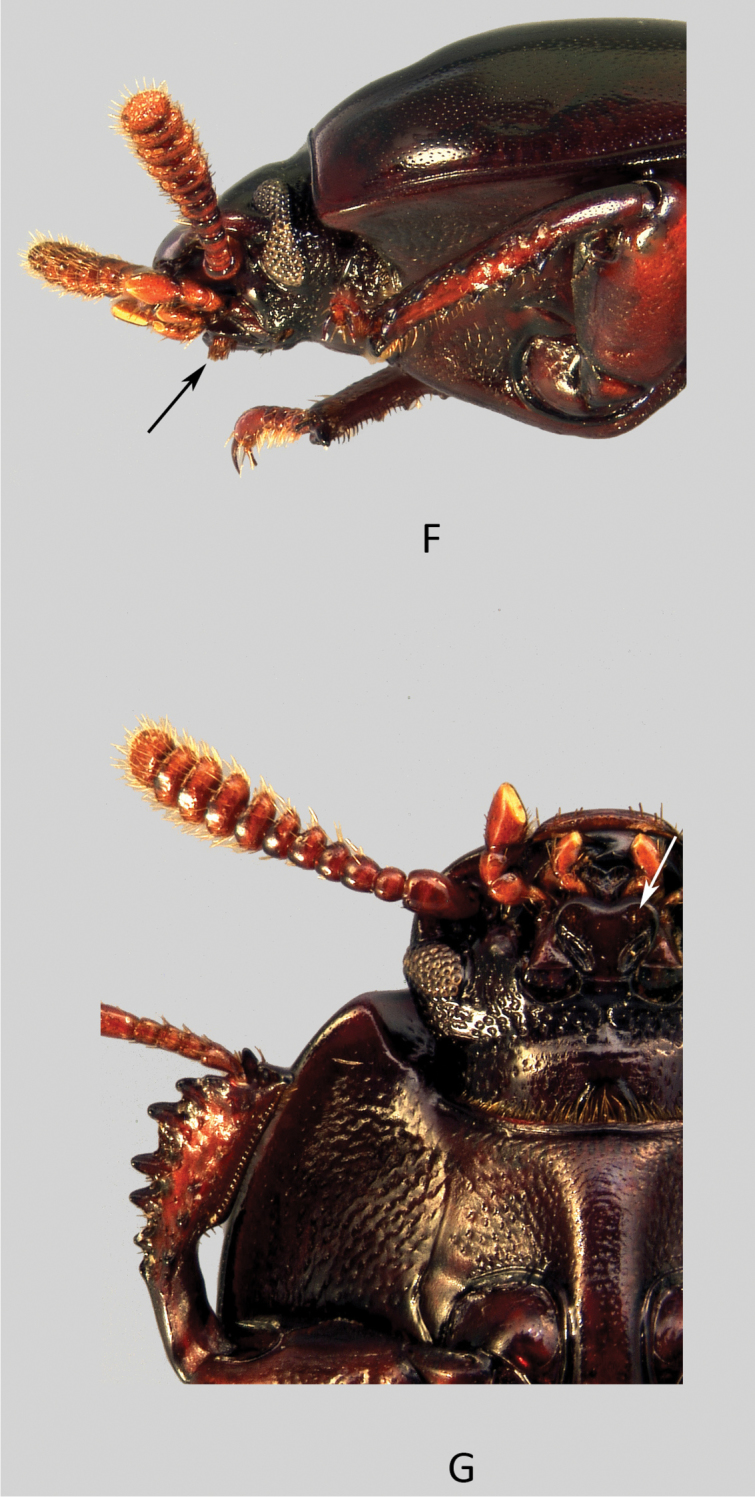
*Uloma condaminei*: **F** forebody (lateral view) **G** forebody (ventral view). The arrows show the apical hair tufts on the mentum.

Figs 3E–F , 5A, B, C, D, E , 6F–G

#### Type specimens.

Holotype male, pinned, with genitalia glued on the same card as the specimen itself. Original label: “Nouvelle-Calédonie, Roches de Ouaième, 2.XI.2010, H. Jourdan & C. Mille leg. / 20°38.333'S, 164°52.092'E ca 680 m alt.” / *Uloma condaminei* m. n. sp. L. Soldati det. 2013, HOLOTYPE ♂ (red printed label) (MNHN); Allotype female. Original label: “Nouvelle-Calédonie, Roches de Ouaième, 2.XI.2010, H. Jourdan & C. Mille leg. / 20°38.283'S, 164°52.010'E, ca 700 m alt.” / *Uloma condaminei* m. n. sp. L. Soldati det. 2013, ALLOTYPE ♀ (red printed label) (MNHN); Paratypes: one male (MNHN), one male and one female (CS): “Nouvelle-Calédonie, Roches de Ouaième, 4.XI.2010, H. Jourdan & C. Mille leg. / 20°38.567'S, 164°51.607'E, ca 800 m alt.” / *Uloma condaminei* m. n. sp. L. Soldati det. 2013; Paratypes: one male (CS), one male (HNHM) one female (CBGP), “Nouvelle-Calédonie, Roches de Ouaième, 4.XI.2010, H. Jourdan & C. Mille leg. / 20°38.333'S, 164°51.947'E, ca 750 m alt.”/ *Uloma condaminei* m. n. sp. L. Soldati det. 2013; Paratype: one male (CBGP) “Nouvelle-Calédonie, Roches de Ouaième, 1.XI.2010, H. Jourdan & C. Mille leg. / 20°38.400'S, 164°52.285'E ca 600 m alt.” / *Uloma condaminei* m. n. sp. L. Soldati det. 2013.

#### Diagnosis.

As underlined beforehand, *Uloma condaminei* is morphologically closely related to *Uloma clamensae* sp. n. It is also morphologically related to *Uloma paniei* Kaszab, 1982 and *Uloma robusta* Kaszab, 1986 with whom it shares a similar type of aedeagus. *Uloma condaminei* can be distinguished from the former two by looking at the pilosity of the mentum. In *Uloma condaminei*, mentum’s pilosity is reduced to two apical hair tufts on each sides (Fig. 6F–G) while in *Uloma paniei* and in *Uloma robusta* the sides of the mentum are completely fringed, from the lateral grooves to the anterior edge. Furthermore, the basal notch at the inner side of the anterior tibiae is larger and deeper (more than one-third of the inner side total length). The average size of *Uloma condaminei* is also smaller (8.0–10.0 mm instead of 10.5–12.2 mm).

#### Description.

Length 8.0–10 mm; width 3.2–4.0 mm. Shining, pitchy dark brown. Antennae, mouthparts, legs and sometimes elytra reddish-brown.

Head: (Fig. 5E) Male: Transverse, genae rounded and continuous in curved line with the clypeus. Frontoclypeal suture not grooved. Frons and clypeus fused in a flat shagreened and dull surface covered with extremely fine, sparse and barely visible punctures. Vertex convex and separated from the frons by a light transverse depression that links the tempora together behind the eyes. Tempora (densely) and vertex (sparsely) coarsely punctured. Female: contrary to the male, the frontoclypeal area is finely punctate and shining and, at the location of the suture, there is a slight curved depression.

Antennae (Fig. 5E) gradually becoming transverse and expanded from antennomere 5. Antennomeres 5–7 flattened with the apical edges more or less lobate and dull.

Mentum (Figs 6F–G) similar to *Uloma clamensae*, cordate, flat, with two oblique lateral grooves near the base and two apical dense hair tufts (Fig. 6F), all arranged symmetrically in relation to midline; disc unpunctured and shining. In the female, the mentum’s shape is rounder, the two oblique lateral grooves are closer, longer and deeper so that the midline appears to be convex and the anterior emargination very light.

Pronotum: about 1.2 times wider than long, sides weakly arcuate, widest around the middle. Rim on the anterior margin disappears completely on a short length in the middle; base unrimmed, with exception of two short folds located at the level of the two concave curves of external margin. Anterior angles 90° but smooth at the top and slightly protruding forward, posterior ones obtuse. Lateral rims becoming progressively thinner from the base toward the anterior angles. Whole upper surface of the pronotum finely and densely punctate, sparser on the disc but denser on the sides.

Male: antero-median depression of pronotum well impressed, not reaching half of pronotal length, its posterior edge arcuate and delimited by four very faint elevations. The lateral bumps anterolaterally bordering the depression low and not projecting to anterior edge. Interior of depression somewhat more strongly punctate than rest of pronotal surface.

Female: pronotum regularly convex, without antero-median depression and overall punctate.

Prosternal process in lateral view obliquely bent beneath procoxae.

Elytra quite convex, humeral angles of lateral margin protruding. Lateral margin barely visible in dorsal view except in the middle. Each elytron bears nine grooved striae of punctures and a faint scutellary striole. Strial punctures are slightly wider than grooves. Elytral intervals nearly flat on disc and becoming slightly convex laterally and toward apex, covered with fine and superficial punctation.

Metaventrite short (Fig. 5C), between meso- and metacoxae about as long as the length of a mesocoxa.

Abdominal ventrites 1–4 (Fig. 5C) finely and densely punctate on a narrow median longitudinal strip. On each side of this longitudinal strip, punctation becomes progressively larger and sparser toward the sides and the integument’s surface is slightly striate longitudinally. The anal ventrite finely and sparsely punctate, its outer margin without rim, except a very short fold on both sides, just in front of the base.

Anterior tibiae (Fig. 5D) with only a faint trace of carina on their upper surface and strongly notched at base of at least one-third of the length of the inner side.

Aedeagus: on tergal face (Fig. 3E), the basal two-third of the parameres are bottleneck-shaped, then suddenly enlarged and truncate at the apex. In lateral view (Fig. 3F), parameres are bisinuate and narrowed toward apex.

#### Etymology.

This new species is named after our friend and colleague Dr. F.L. Condamine who was a PhD student at the time we prospected in New Caledonia. He is also a member of the “All Blaps” team.

#### Distribution.

*Uloma condaminei* is currently known only from New Caledonia where it is endemic.

### Uloma isoceroides

(Fauvel, 1904)

http://species-id.net/wiki/Uloma_isoceroides

Figs 2B , 3G–H

Melasia isoceroides Fauvel, Revue d’Entomologie 23: 180, 182.
Uloma isoceroides Fauvel, Gebien H. 1911, Tenebrionidae III. In: Schenkling S (Ed) *Coleopterorum Catalogus*. Vol. 18: 402.

#### Type locality.

Baie du Prony, Mont Mou, Ourail, Kanala.

#### Type specimens.

Lectotype male and paralectotypes (designated by Kaszab 1982) males and females of *Melasia isoceroides* Fauvel (IRSNB), none examined.

#### Diagnosis.

*Uloma isoceroides* is one of the four species of the group in which the mentum of the male is completely glabrous and flat. It can be separated from *Uloma caledonica* by the shorter metaventrite, (between meso- and metacoxae hardly longer than half of the length of a mesocoxa) and the humeri not developed. It differs from *Uloma jourdani* by the outer margin of terminal ventrite (anal sternite) regularly arcuate, without lateral sinuosities, the mentum as long as broad or longer, not cordate. Moreover, all the male antenomeres are shining and the aedeagus is different. It differs also from *Uloma kergoati* by the elytral striae of punctures normally marked and developed to the apex, the pronotum quite densely and sharp punctate, and the different aedeagus. Its size is also smaller in average (7.0-8.8 mm). Aedeagus (Fig. 3G, H) similar to the one of *Uloma caledonica* (with the parameres bottleneck-shaped) but truncate (not notched) at the apex.

#### Distribution.

Kaszab (1982: 86) cited this species from the following localities: Baie du Prony, Mt Mou, Ourail, Mt Rembai, Mt Do, Kanala [Canala], Plaine des Lacs, Pic du Pin, Rivière Bleue, Mt Koghi, Nouméa. “Neukaledonien (Grande Terre, Zentral Massiv und SW”.

#### Additional localities.

Monts Koghis (22°10.63'S, 166°30.49'E, ca 460 m) 4.III.2008, L. Soldati, G.J. Kergoat & H. Jourdan rec. (CBGP); Réserve Botanique de Bois du Sud (22°10.41'S, 166°45.83'E, ca 210 m) 8.III.2008, L. Soldati, G.J. Kergoat & H. Jourdan rec. (CBGP); Plateau de Dogny (21°37.03'S, 165°53.05'E, ca 920 m) 29.X.2008, L. Soldati, G.J. Kergoat & F.L. Condamine rec. (CBGP); Massif forestier de la Tchamba (21°00.71'S, 165°15.58'E, ca 200 m) 8.IV.2009, L. Soldati, G.J. Kergoat, H. Jourdan & F.L. Condamine rec. (CBGP).

#### Discussion:

As underlined by the results of the PTP molecular species delimitation analyses, there is potentially some level of cryptic diversity for this species. One putative species corresponds to the material collected in the Plateau de Dogny, whereas the other putative species corresponds to material collected in the Tchamba forest mountain range. Further studies based on a larger sampling from additional localities should clarify this finding and possibly discern one or more cryptic species.

### Uloma jourdani

L. Soldati sp. n.

http://zoobank.org/390037E3-3B06-48F9-A784-0A23B2117BC8

http://species-id.net/wiki/Uloma_jourdani

**Figure 7.**
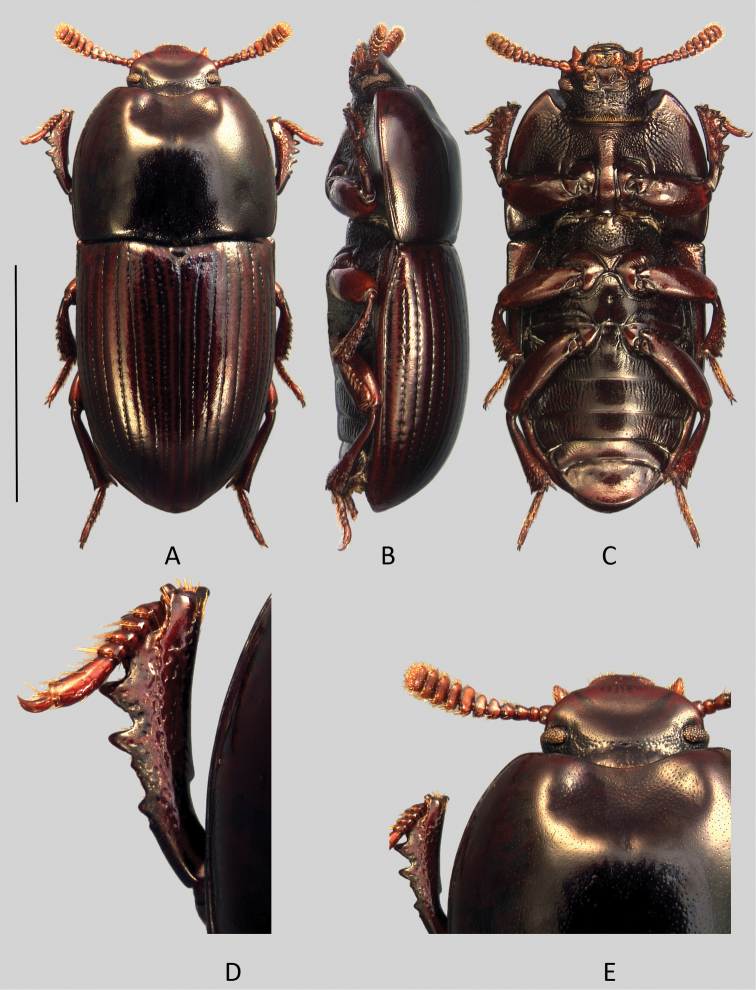
*Uloma jourdani*: **A** habitus (dorsal view) **B** habitus (lateral view) **C** habitus (ventral view) **D** anterior tibia (upper face) **E** head (dorsal view). Scale bar: 5 mm.

Figs 3I–J , 7A, B, C, D, E

#### Type specimens.

Holotype male, pinned, with genitalia glued on the same glue board as the specimen itself. Original label: “Nouvelle-Calédonie, Massif du Panié, Dawenia, 13.XI.2010, Jourdan & Mille rec. / 20°32.268'S, 164°40.903'E, ca 640 m NC130-2a’” / Uloma jourdani m. n. sp. L. Soldati det. 2013, HOLOTYPE ♂ (red printed label) (MNHN); Allotype female, pinned. Original label: “Nouvelle-Calédonie, Massif du Panié, Dawenia, 14.XI.2010, H. Jourdan & C. Mille / 20°32.290'S, 164°40.967'E, ca 620 m NC139-2a’” / Uloma jourdani m. n. sp. L. Soldati det. 2013, ALLOTYPE ♀ (red printed label) (MNHN); Paratypes: one male (MNHN) and one female (CBGP): “Nouvelle-Calédonie, Massif du Panié, Dawenia, 13.XI.2010, H. Jourdan & C. Mille / 20°32.268'S, 164°40.903'E, ca 630 m; Paratypes: two males (CBGP): “Nouvelle-Calédonie, Massif du Panié, Dawenia, 13.XI.2010, H. Jourdan & C. Mille / 20°32.268'S, 164°40.903'E, ca 640 m”; Paratypes: one male (CS): “Nouvelle-Calédonie, Massif du Panié, Dawenia, 12.XI.2010, H. Jourdan & C. Mille / 20°32.265'S, 164°40.843'E ca 620 m”; Paratypes: one male and one female (CS): “Nouvelle-Calédonie, Massif du Panié, Dawenia, 14.XI.2010, H. Jourdan & C. Mille / 20°32.262'S, 164°41.092'E ca 620 m”; Paratype: one female (CS): “Nouvelle-Calédonie, Massif du Panié, Dawenia, 14.XI.2010, H. Jourdan & C. Mille / 20°32.290'S, 164°40.967'E ca 620 m”.

#### Other material.

one male, Nouvelle-Calédonie, Massif du Panié, Wewec, forêt sur pente, 20°35.63'S, 164°43.66'E ca 420 m, 8.XI.2010, H. Jourdan & C. Mille rec.; one female, Massif du Panié, La Guen, 20°37.48'S, 164°46.83'E ca 580 m, 23.XI.2010, H. Jourdan & C. Mille rec.; one female, Massif du Panié, La Guen, 20°37.50'S, 164°46.83'E ca 590 m, 19.XI.2010, H. Jourdan & C. Mille rec.; two males and one female, Massif du Panié, La Guen, 20°37.50'S, 164°46.83'E ca 590 m, 18-25.XI.2010, H. Jourdan & C. Mille rec.; one male, Massif du Panié, La Guen, 20°37.50'S, 164°46.92'E ca 570 m, 18.XI.2010, H. Jourdan & C. Mille rec.

#### Diagnosis.

The completely glabrous and flat mentum of *Uloma jourdani* males is also found in *Uloma caledonica*, *Uloma isoceroides* and *Uloma kergoati*. *Uloma jourdani* can be distinguished from *Uloma caledonica* by its shorter metaventrite (the part between meso- and metacoxae hardly longer than half of the length of a mesocoxa), by the reduced humeri and also by different male aedeagus. It differs from *Uloma isoceroides* and *Uloma kergoati* by the shape of the terminal ventrite (anal sternite), by the presence of a dull shagreened patch on the upper face of male antennomeres 5–7 and also by differences in male aedeagus.

#### Description.

Length 8.0–9.0 mm; width 4.0–4.2 mm. Shining, pitchy dark brown, elytra often brighter, dark red-brown. Antennae, mouthparts, legs and elytra reddish-brown.

Head (Fig. 7E).

Male: Transverse, genae straight just in front of the eyes, then continuous in curved line with the clypeus. Frontoclypeal suture shallowly impressed. Frons and clypeus fused in a shagreened and dull surface covered with extremely fine, sparse and barely visible punctures. Vertex convex, shining and separated from the frons by a deep transverse impression that extends behind the eyes. Tempora and vertex (more sparsely) coarsely punctured.

Female: contrary to the male, the frontoclypeal area is finely and densely punctate over a shining background. The frontoclypeal junction is slightly convex and there are two feebly impressed oblique lateral lines at the place of the clypeogenal suture. In between, the transversal line of the suture is barely visible.

Antennae (Fig. 7E) gradually becoming transverse and expanded from antennomere 5. Antennomeres 5–9 flattened with the apical edges more or less protruding in the middle, especially the 7^th^. In the males, antennomeres 5-7 are dull and shagreened on their upper face only.

Mentum (Fig. 7C) transverse, cordate, flat, with two oblique lateral grooves arranged symmetrically in relation to midline; disc flat, covered with a dense, extremely fine and horizontally confluent punctation. In the female, the mentum is similar to the male’s one, but the punctation is less dense and distinct.

Pronotum: about 1.3 times wider than long. Sides narrow in light curve from rear to front, widest just in front of the base. Rim on the anterior margin obliterates completely in the middle; base unrimmed, with exception of two very short folds located at the level of the two concave curves of external margin. Anterior angles 90°but smooth at the top and slightly protruding forward, posterior ones obtuse. Lateral rims becoming progressively thinner from the base toward the anterior angles. Whole upper surface of the pronotum finely punctate, sparser on the disc but denser on the sides.

Male: antero-median depression of pronotum well impressed, quite broad, not reaching half of pronotal length, its posterior edge arcuate and delimited by four very faint elevations. The lateral bumps anterolaterally bordering the depression’s sides forward are low.

Female: pronotum regularly convex, without antero-median depression and overall finely punctate, but denser on the sides.

Prosternal process in lateral view in steep slope beneath procoxae.

Elytra convex, slightly oval, sides not subparallel. Humeral angles of lateral margin feebly protruding and generally covered by the posterior angles of pronotum. Lateral margin invisible in dorsal view, except at the level of the humeral angles and at the rear of elytra. Each elytron bears nine grooved striae of punctures and a faint scutellary striole. Strial punctures are slightly wider than grooves. Elytral intervals flat on disc and becoming very slightly convex laterally - but not at the apex - covered with fine and superficial punctuation.

Metaventrite short, between meso- and metacoxae, about half the length of a mesocoxa.

Anterior tibiae (Fig. 7D) with only a faint trace of carina on their upper surface and strongly notched at base of at least one-fourth of the length of the inner side.

Aedeagus: on tergal face (Fig. 3I), the basal two-third of the parameres are bottleneck-shaped, then slightly enlarged and securiform at the apex. In lateral view (Fig. 3J), parameres are bisinuate and narrowed toward apex.

#### Etymology.

This new species is named after our friend Dr. H. Jourdan (IRD Nouméa) great connoisseur of New Caledonia. He is also a member of the “All Blaps” team.

#### Distribution.

At present, *Uloma jourdani* is only known from the surroundings of Dawenia, in a valley situated at the foot of the western slopes of Mount Colnett in New Caledonia.

#### Discussion.

As underlined by the results of the PTP molecular species delimitation analyses, there is potentially some level of cryptic diversity for this species. One putative species correspond to the material collected in Dawenia (in the Panié mountain range), whereas the other putative species correspond to material collected in La Guen and Wewec (in the Panié mountain range). Both groups are apparently morphologically indistinguishable, but we cannot exclude the possibility that future studies may find some morphological differences between the two. To avoid complicating possible future taxonomic revisions, we chose to only select specimens from one of the two putative groups (i.e. the specimens collected in Dawenia) as reference for all the type material.

### Uloma kergoati

L. Soldati sp. n.

http://zoobank.org/A06836E0-2321-44B0-8828-8049C9EA7AAD

http://species-id.net/wiki/Uloma_kergoati

**Figure 8.**
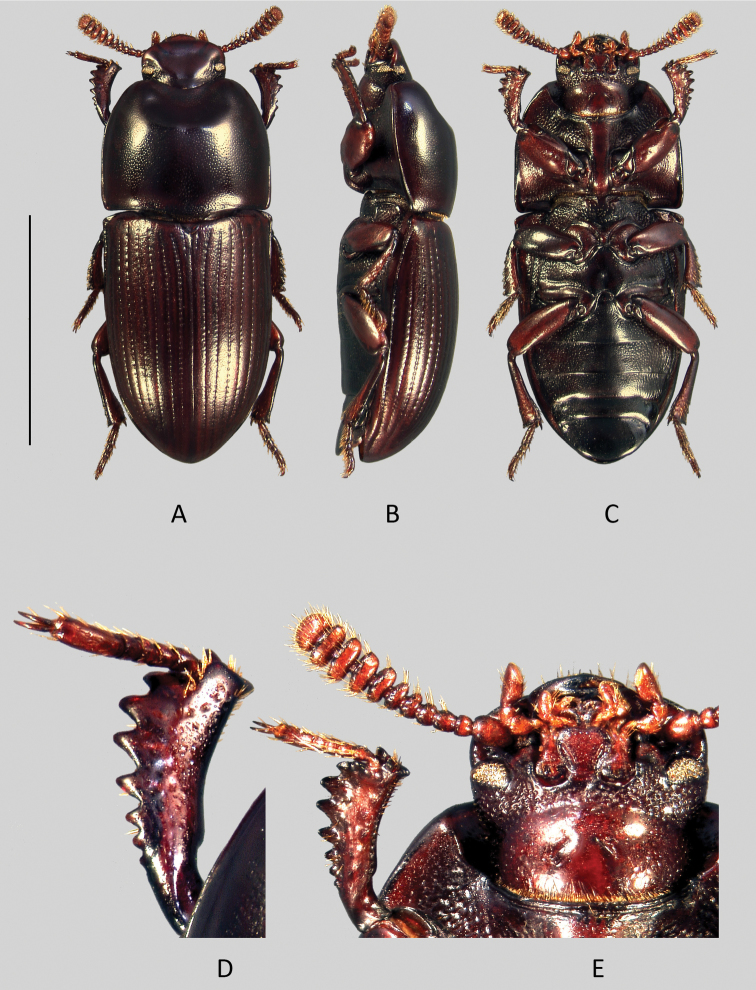
*Uloma kergoati*: **A** habitus (dorsal view) **B** habitus (lateral view) **C** habitus (ventral view) **D** anterior tibia (upper face) **E** head (dorsal view). Scale bar: 5 mm.

Figs 3K–L , 8A, B, C, D, E

#### Type specimens.

Holotype male, pinned, with genitalia glued on the same card as the specimen itself. Original label: “Nouvelle-Calédonie, Massif du Kouakoué, 17-23.III.2008, H. Jourdan, G. Kergoat & L. Soldati leg. / 21°57.427'S, 166°32.294'E, ca 1280 m alt. / *Uloma kergoati* m. n. sp. L. Soldati det. 2013, HOLOTYPE ♂” (red printed label) (MNHN); Allotype female, pinned. Original label: “Nouvelle-Calédonie, Massif du Kouakoué, 17-23.III.2008, H. Jourdan, G. Kergoat & L. Soldati leg. / 21°57.427'S, 166°32.294'E, ca 1280 m alt. NC16-2b” / *Uloma kergoati* m. n. sp. L. Soldati det. 2013, ALLOTYPE ♀ (MNHN); Paratypes, same data as holotype: one female (MNHN), one male (HNHM), two males (CBGP), three males and one female (CS).

#### Diagnosis.

The completely glabrous and flat mentum of *Uloma kergoati* males is also found in *Uloma caledonica*, *Uloma isoceroides* and *Uloma jourdani*. It differs from *Uloma caledonica* by its shorter metaventrite (hardly longer than half of the length of a mesocoxa), by the reduced humeri and also by differences in male aedeagus. It can easily be distinguished from *Uloma jourdani* by the shining surface of the upper face of all male antennomeres and the aedeagus. It also differs from *Uloma isoceroides* by the elytral striae of punctures that become finer and blurred toward apex; in addition, the male aedeagus of these two species are also very distinctive.

#### Description.

Length 8.0–11 mm; width 3.8–4.2 mm. Shining, pitchy dark brown. Antennae, mouthparts, legs and elytra reddish-brown.

Head (Fig. 8E).

Male: Transverse, genae straight in front of the eyes, then continuous in curved line with the clypeus. Frontoclypeal suture faintly impressed. Frons and clypeus fused in a flat shagreened and dull surface covered with extremely fine, sparse and barely visible punctures. Vertex separated from the frons by a superficial transverse impression. Tempora coarsely punctured. Vertex with very fine and obsolescent punctures, the background dull like the frontoclypeal area.

Female: contrary to the male, the frontoclypeal area is finely punctate and shining and, at the location of the suture, there is a shallow curved depression.

Antennae (Fig. 8E) gradually becoming transverse and expanded from antennomere 5. Antennomeres 5–9 flattened with the apical edges more or less protruding.

Mentum (Fig. 8E) cordate, flat, with two oblique divergent lateral grooves near the base. In the female, the mentum is narrower, the two oblique lateral grooves are closer, larger and less oblique (i.e. more parallel), the anterior margin is truncate.

Pronotum. Male: about 1.2 times wider than long, sides nearly straight in the basal half, then regularly arcuate toward the anterior angles, widest in front of the middle. Rim on the anterior margin disappears in the middle at level of the antero-median depression; at the same place, the anterior margin is emarginate and concave. Base without rim, except two very short folds located at the level of the two concave curves of external margin. Anterior angles 90°, posterior ones slightly obtuse. Whole upper surface of the pronotum densely punctate, sparser on the disc but denser and finer on the sides. Antero-median depression of pronotum quite deep, not reaching half of pronotal length, its posterior edge arcuate with a slight median impression. Interior of antero-median depression more coarsely punctate than rest of pronotal surface, the ground dull and shagreened.

Female: regularly convex, without antero-median depression and overall sharply and densely punctate, the punctures finer on the sides. Pronotum widest at base, then narrowed toward the front; the anterior edge tri-sinuate.

Prosternal process in lateral view obliquely bent beneath procoxae.

Elytra. Elytra quite convex transversally, humeri reduced. Humeral angles of lateral margin protruding and divergent (especially in the males); sides subparallel on one-third of the basal part, then regularly acuminate. Lateral margin visible in dorsal view except at level of ventrites 1-2. Each elytron bears nine grooved striae of punctures that tend to obliterate at the apex and a scutellary striole. Strial punctures are slightly wider than grooves. Elytral intervals nearly flat, covered with fine punctuation on a shining ground.

Metaventrite short, between meso- and metacoxae about as long as half the length of a mesocoxa.

Abdomen. Abdominal ventrites 1–4 (Fig. 8C) finely and densely punctate on a narrow median longitudinal strip. On each side of this longitudinal strip, the punctation becomes progressively larger and sparser toward the sides before mixing up with longitudinal striae, except on the 4^th^ ventrite where the striae are less developed. The anal ventrite finely punctate, sparsely toward the sides, its outer margin without rim.

Legs. Anterior tibiae (Fig. 8D) without carina on their upper surface and strongly notched at base of about one fourth of the inner side length.

Aedeagus. On tergal face (Fig. 3K), basal two-third of the parameres are bottleneck-shaped, then suddenly enlarged and arcuate at the apex, with two lateral teeth on each side. In lateral view (Fig. 3L), parameres are bisinuate and narrowed toward apex.

#### Etymology.

This new species is named after Dr. G.J. Kergoat researcher at the CBGP, member of the “All Blaps” team and one of the “survivors” of the Kouakoué expedition.

#### Distribution.

*Uloma kergoati* is currently known only from New Caledonia where it is endemic.

### Uloma monteithi

Kaszab, 1986

http://species-id.net/wiki/Uloma_monteithi

Figs 2C , 3M–N

Uloma monteithi Kaszab, Annales Historico-Naturales Musei Nationalis Hungarici, 78: 160.

#### Type locality.

Aoupinié, 20 km NE Poya.

#### Type specimens.

Holotype male. Original label: “NEW CALEDONIA, Aoupinié, 20 km NE Poya, 650 m, 18–19 May 1984, G. Monteith & D. Cook / Queensland Museum, Brisbane, Reg. N°T.10111 / Holotypus 1986 ♂ *Uloma monteithi* Kaszab” (QM); Paratypes (same data as Holotype): one female (QM) and one male (Hnhm), all examined.

#### Diagnosis.

Among the *Uloma isoceroides* species group, *Uloma monteithi* can easily be distinguished by the mentum which is concave along the longitudinal axis (flat in all the other species of this group), shining, unpunctured. Male anterior tibiae strongly notched at base up to nearly half of the length of the inner face, then they extend straight to the apex. Pronotum upper surface finely punctate, sparser on the disc and denser on the sides. Elytra sharply striate-punctate, distinctly shallower at the apex. Elytral intervals quite flat, covered with extremely fine punctures, the background smooth and shining. Humeri not developed, metaventrite short like in *isoceroides*, wings reduced, flightless. Aedeagus (Fig. 3M–N). Length: 8.2-9.0 mm.

#### Distribution.

So far, only known from the type locality.

### Uloma opacipennis

(Fauvel, 1904)

http://species-id.net/wiki/Uloma_opacipennis

Figs 2D , 3O–P

Melasia opacipennis Fauvel, Revue d’Entomologie 23: 180, 182.
Uloma opacipennis Fauvel, Gebien H. 1911, Tenebrionidae III. In: Schenkling S (Ed) *Coleopterorum Catalogus*. Vol.18: 403.

#### Type locality.

Baie du Prony, Nouméa.

#### Type specimens.

Lectotype male of *Melasia opacipennis* Fauvel (IRSNB); Paralectotypes: two females of *Melasia opacipennis* Fauvel (IRSNB), none examined. Lectotype and Paralectotypes designated by Kaszab (1982).

#### Diagnosis.

*Uloma opacipennis* can be distinguished morphologically from all other New Caledonian species by the structure of its elytra, the integument of which is dull and shagreened, by the presence of a tooth on the underside of the head capsule on the postgenal margin, by the glabrous mentum of the male whose disc is convex between the two lateral subparallel grooves which are long and nearly reach the anterior edge, and by its characteristic aedeagus (Fig. 3O, P). Elytral striae crisp. Striae 1-3 thinner and shallower on the apical declivity. Rows of punctures dense and slightly wider than the striae. Antero-median depression of the pronotum in the male small and rounded. Pronotum finely and sparcely punctate. Male anterior tibiae slightly notched at base on the internal face. Male antennae distinctly expanded from 5^th^ antennomere. Average size small: 7.0–7.5 mm long.

*Uloma opacipennis* is morphologically unrelated to the other species of the *Uloma isoceroides* group. That said, molecular phylogenetic analyses indicate that it is a member of the same evolutionary lineage, hence its inclusion in the species group. On a morphological point of view, all the species of the *Uloma isoceroides* group, except *Uloma opacipennis*, share the following characters: Head short and broad. Male with clypeus and frons located in the same plane, not impressed along the clypeofrontal suture, flat, with a shagreened dull surface covered with extremely fine, sparce and barely visible punctation. Metaventrite short, between median and posterior coxae approximately as long as or hardly longer than half of the length of a median coxa. Humeri slightly developed or reduced. Flightless species. On the contrary, in *Uloma opacipennis* the male head is normal, i. e. impressed along the clypeofrontal suture, not flattened and its surface is distinctly punctate. Metaventrite long, between median and posterior coxae longer than a median coxa. Humeri developed. Fully winged.

#### Distribution.

Kaszab (1982: 95) cited this species from the following localities: Mt Panié, 250 m; Houadou (Karovin, Houailou) Riv.; Col d’Amieu, 500 m; Montagne des Sources; Rivière Bleue; Mt Koghi, 450–600 m; Îles Loyauté: Lifou, Wu. “Neukaledonien (Grande Terre von NW bis SO); Loyauté (Lifou)”.

#### Additional localities.

Mont Koghis (22°10.63'S, 166°30.49'E, ca 460 m alt.) 4.III.2008, L. Soldati, G.J. Kergoat & H. Jourdan rec. (CBGP); Mts Koghis, ca 400 m, 26 may 1984, G. Monteith & D. Cook (QM); Réserve botanique de Bois du Sud (22°10.41'S, 166°45.83'E, ca 210 m) 8.III.2008, L. Soldati, G.J. Kergoat & H. Jourdan rec. (CBGP); Parc des Grandes Fougères, Pic Vincent (21°36.16'S, 165°46.44'E, ca 690 m) 28.III.2008, L. Soldati, G. Kergoat & H. Jourdan rec. (CBGP); Réserve de Yaté Barrage (22°09.23'S, 167°53.51'E, ca 270 m) 23.X.2009, L. Soldati, G.J. Kergoat, F.L. Condamine & H. Jourdan rec. (CBGP); Roches de Ouaième (20°38.28'S, 164°52.01'E, ca 700 m) 2.XI.2010, H. Jourdan & C. Mille rec. (CBGP); Massif du Panié, La Guen (20°37.50'S, 164°46.83'E, ca 590 m) 18-25.XI.2010, H. Jourdan & C. Mille rec. (CBGP); Massif du Panié, La Guen (20°37.42'S, 164°46.85'E, ca 590 m) 20.XI.2010, H. Jourdan & C. Mille rec. (CBGP); Massif du Panié, Dawenia (20°32.26'S, 164°40.90'E, ca 630 m) 15.XI.2010, H. Jourdan & C. Mille rec. (CBGP).

### Uloma paniei

Kaszab, 1982

http://species-id.net/wiki/Uloma_paniei

Fig. 2E

Uloma paniei Kaszab, Folia Entomologica Hungarica 18: 84.

#### Type locality.

Mont Ignambi.

#### Type specimens.

Holotype male: “Nouvelle-Calédonie, Mt Ignambi, 2100 ft, 7.VIII.1914, leg. P. D. Montague” (BMNH); Paratypes: Mt Panie, 1911, P. D. Montague (one male and one female, BMNH); Ignambi Gipfel, 1300 m, 15.IV.1911, leg. F. Sarasin & J. Roux (one male, MTD); Panie Wald, 500 m, 27.VI.1911, leg. F. Sarasin & J. Roux (one female, MTD); Mt Panier [misspelled], 1200 m, 9.X.1967, leg. J. & M. Sedlacek (two females, BPBM). None examined.

#### Diagnosis.

Within the *Uloma isoceroides* species group, *Uloma paniei* and *Uloma robusta* are the only species whose mentum of the male is adorned with two peripheral hair fringes along the sides and the front edge, leaving the disc glabrous. Both species have the male anterior tibiae shortly notched at base, maximum one third of the length of inner face. Size large (10.5-12.2 mm). *Uloma paniei* may be separated from *Uloma robusta* by the male anterior tibiae strongly and deeply notched at base of the inner face (up to one third of the inner side length), the disc of the mentum smooth and shining between the peripheral hair fringes in the males, the elytral surface shining, the striae deeper and expanded to the apex. The male aedeagus is similar in both species. It is unfortunately impossible to identify the females on the basis of morphological characters.

#### Distribution.

Kaszab (1982: 84) cited this species from the following localities: Mt Ignambi, Mt Panié. “Neukaledonien (Grande Terre NW)”.

#### Additional localities.

Mt Panié, 450–950 m, 14 May 1984, G. Monteith & D. Cook (QM).

### Uloma robusta

Kaszab, 1986

http://species-id.net/wiki/Uloma_robusta

Figs 2F , 3Q–R

Uloma robusta Kaszab, Annales Historico-Naturales Musei Nationalis Hungarici 78: 159.

#### Type locality.

Mont Panié.

#### Type specimens.

Holotype male. Original labels: “NEW CALEDONIA, Mt Panié, 1300–1600 m, 15 May 1984, G. Monteith & D. Cook / Queensland Museum, Brisbane, Reg. N°T.10108 / Holotypus 1986 ♂ *Uloma robusta* Kaszab” (QM); (QM); Paratypes (same data as Holotype): three females (QM) and one male (Hnhm), all examined.

#### Diagnosis.

*Uloma robusta* closely resembles *Uloma paniei* and both species occur in the same area of the northeastern mountain range of New Caledonia. However, in *Uloma robusta* the male anterior tibiae are less strongly notched at base of the inner face (about one-fifth of the inner side length), the disc of the mentum is coarsely punctate between the peripheral hair fringes, except on a narrow mid-longitudinal strip, the elytral surface is shagreened and dull and the striae shallower with a tendency to obliterate toward apex (especially striae 2, 3, 6 and 7). In *Uloma paniei*, on the contrary, the disc of the mentum is smooth and shining between the peripheral hair fringes, the elytral surface shining, the striae deeper and clearly visible up to the apex. The male aedeagus is similar in both species. It is unfortunately impossible to identify the females on the basis of morphological characters.

#### Distribution.

*Uloma robusta* is probably endemic to the Panié mountain range.

#### Discussion.

*Uloma robusta* is possibly a junior synonym of *Uloma paniei*. However, it was not possible for us to test this hypothesis based on the material we examine.

## Discussion

### Integrative taxonomy

The use of a combined approach based on morphology and on molecular data allowed us to better circumscribe the boundaries within a morphologically homogeneous group of species and to define the characteristics of the *Uloma isoceroides* species group. Without the results of molecular phylogenetic analyses, it would have been impossible to determine that *Uloma opacipennis* is a member of the same evolutionary lineage. The fact that *Uloma opacipennis* is in a derived position within the group also allow us to hypothesize that this taxon secondarily developed unique attributes of its own (elytra and head structures, shape of the aedeagus). The analyses of molecular species delimitation also provide more evidence to support the species status of the newly described species. It is especially the case for *Uloma clamensae* and *Uloma condaminei*, two species that are morphologically very close. In addition, the PTP analyses suggest some unsuspected cryptic biodiversity for two species (*Uloma jourdani* and *Uloma isoceroides*). For *Uloma isoceroides*, the fact that only two specimens were sequenced does not really allow us to confirm this hypothesis because of possible geographical sampling biases (Bergsten et al. 2012). On the contrary the sampling for *Uloma jourdani* is denser and the results are likely not artefactual. The two potential species *Uloma jourdani* clusters also have a disjunct distribution: members of the largest molecular group (six individuals) were only collected in Dawenia (in the Panié mountain range) while the members of the smallest cluster (four individuals) were collected in La Guen and Wewec (also in the Panié mountain range, separated by less than 10 km). Because members of both clusters are completely morphologically indistinguishable (even the males) we did not chose to describe two species. That said – as underlined in the results section – in the description of *Uloma jourdani* we chose to only use representatives of one cluster (the one from Dawenia) to provide type material. Alternatively we could have followed the views of several authors (e.g. Jörger and Schrödl 2013) who propose to use DNA sequence information as a line of evidence to describe cryptic diversity. Though we agree that this approach is another way of describing diversity, we prefer to remain conservative, pending the eventual discovery of diagnostic morphological characters.

## Conclusions

The tenebrionid fauna of New Caledonia is rich and diverse with a level of high endemism: of the 238 species (including the four new species described here), 219 (92%) are unique to New Caledonia. By applying our integrative approach to a broader sampling of *Uloma* or to other tenebrionid genera, we expect to discover new species in the genus *Uloma* but also in the well-diversified genera *Isopus* Montrouzier, 1860 (Cnodalonini, 35 described species, Kaszab 1982, 1986) and *Callismilax* F. Bates, 1874 (Titaenini, 51 described species, Kaszab 1982, 1986). Such a high level of taxonomic endemism is not uncommon for several clades that diversiﬁed in New Caledonia; e.g. 94% of the New Caledonian cricket fauna is endemic to the archipelago (Robillard and Desutter-Grandcolas 2008). In addition to the high endemism, the genus *Uloma* is of particular interest for the New Caledonian archipelago because it harbours a species diversity that is comparable to Australia (Australia has 27 species of *Uloma* while New Caledonia has now 26 species). In New Caledonia, despite the fact that most of *Uloma* species are wingless, they have been able to colonize very distinct lowland and mountainous ecosystems (cloud forest, dry forests, evergreen forests, maquis). Some *Uloma* species appear to have allopatric distributions but sympatric distributions seem to be the predominant pattern (Kaszab 1982, 1986). Personal observations during fieldwork confirmed that up to four species could live in the same rotten trunk. The distribution pattern for *Uloma* spp. can be qualiﬁed as microendemic because single mountains or specific mountain ranges usually harbour typical species communities. This is best shown in the Mont Panié range where at least five species are known to live sympatrically (potentially six). Although the factors that have promoted such an extraordinary pattern of microendemism are still to be determined, we think that future phylogenetic-based analyses coupled with biogeographic and diversification inferences may bring answers to this issue (see for instance the study Condamine et al. 2013 on another group of darkling beetles).

## Supplementary Material

XML Treatment for Uloma caledonica

XML Treatment for Uloma clamensae

XML Treatment for Uloma condaminei

XML Treatment for Uloma isoceroides

XML Treatment for Uloma jourdani

XML Treatment for Uloma kergoati

XML Treatment for Uloma monteithi

XML Treatment for Uloma opacipennis

XML Treatment for Uloma paniei

XML Treatment for Uloma robusta

## References

[B1] Balke M Pons J Ribera I Sagata K Vogler AP (2007a) Infrequent and unidirectional colonization of hyperdiverse *Papuadytes* diving beetles in New Caledonia and New Guinea. Molecular Phylogenetics and Evolution 42: 5005–516 10.1016/j.ympev.2006.07.019 16979911

[B2] Balke M Alarie Y Ribera I Wewalka G (2007b) Molecular Phylogeny of Pacific Island Colymbetini: radiation of New Caledonian and Fijian species. Zoologica Scripta 36: 173–200. doi: 10.1111/j.1463-6409.2006.00265.x

[B3] Bartish IV Swenson U Munzinger J Anderberg AA (2005) Phylogenetic relationships among New Caledonian Sapotaceae (Ericales): molecular evidence for generic polyphyly and repeated dispersal. American Journal of Botany 92: 667–673. doi: 10.3732/ajb.92.4.667 21652444 10.3732/ajb.92.4.667

[B4] Belshaw R Quicke DLJ (2002) Robustness of ancestral state estimates: evolution of life history strategy in ichneumonoid parasitoids. Systematic Biology 51: 450–477. doi: 10.1080/10635150290069896 12079644 10.1080/10635150290069896

[B5] Bergsten J Bilton DT Fujisawa T Elliott M Monaghan MT Balke M Hendrich L Geijer J Herrmann J Foster GN Ribera I Nilsson AN Barraclough TG Vogler AP (2012) The effect of geographical scale of sampling on DNA barcoding. Systematic Biology 61: 851–869. doi: 10.1093/sysbio/sys037 22398121 10.1093/sysbio/sys037 PMC3417044

[B6] Bouchet P Jaffré T Veillon J-M (1995) Plant extinction in New Caledonia: protection of sclerophyll forests urgently needed. Biodiversity and Conservation 4: 415–428. doi: 10.1007/BF00058425

[B7] Bouchet P Jaffré T Veillon J-M (1998) Threatened plants of New Caledonia: Is the system of protected areas adequate? Biodiversity and Conservation 7: 109–135

[B8] Chazeau J (1993) Research on New Caledonian terrestrial fauna: achievements and prospects. Biodiversity Letters 1: 123–129. doi: 10.2307/2999756

[B9] Cluzel D Aitchison JC Picard C (2001) Tectonic accretion and underplating of mafic terranes in the Late Eocene intraoceanic fore-arc of New Caledonia (Southwest Pacific): geodynamic implications. Tectonophysics 340: 23–59. doi: 10.1016/S0040-1951(01)00148-2

[B10] Condamine FL Soldati L Clamens A-L Rasplus J-Y Kergoat GJ (2013) Diversification patterns and processes of wingless endemic insects in the Mediterranean Basin: historical biogeography of the genus *Blaps* (Coleoptera: Tenebrionidae). Journal of Biogeography 40: 1899–1913

[B11] Cruaud A Jabbour-Zahab R Genson G Ungricht S Rasplus J-Y (2012) Testing the emergence of New Caledonia: fig wasp mutualism as a case study and a review of evidence. PLoS ONE 7: . doi: 10.1371/journal.pone.0030941 10.1371/journal.pone.0030941 PMC3285151 22383982

[B12] Deuve T Cruaud A Genson G Rasplus J-Y (2012) Molecular systematics and evolutionary history of the genus *Carabus* (Col. Carabidae). Molecular Phylogenetics and Evolution 65: 259–275. doi: 10.1016/j.ympev.2012.06.015 22750112 10.1016/j.ympev.2012.06.015

[B13] Edgar RC (2004) MUSCLE: multiple sequence alignment with high accuracy and high throughput. Nucleic Acid Research 35: 1792–1797. doi: 10.1093/nar/gkh340 10.1093/nar/gkh340 PMC390337 15034147

[B14] Espeland M Johanson KA (2008a) Revision of the New Caledonian *Hydrobiosella* (Trichoptera: Philopotamidae) with description of ﬁve new species. In: Proceedings of the XIIth International Symposium of Trichoptera, 91–102

[B15] Espeland M Johanson KA (2008b) New species and descriptions of females of the New Caledonian endemic genus *Xanthochorema* (Trichoptera, Hydrobiosidae). In: Grandcolas P (Eds) Zoologia Neocaledonica 6, Systematics and Biodiversity in New Caledonia.Mémoires du Muséum National d’Histoire Naturelle 197: 79–97

[B16] Espeland M Johanson KA Hovmöller R (2008) Early *Xanthochorema* (Trichoptera, Insecta) radiations in New Caledonia originated on ultrabasic rocks. Molecular Phylogenetics and Evolution 48: 904–917. doi: 10.1016/j.ympev.2008.06.006 18620067 10.1016/j.ympev.2008.06.006

[B17] Espeland M Johanson KA (2010) The diversity and radiation of the largest monophyletic animal group on New Caledonia (Trichoptera: Ecnomidae: *Agmina*). Journal of Evolutionary Biology 23: 2112–2122. doi: 10.1111/j.1420-9101.2010.02072.x 20722893 10.1111/j.1420-9101.2010.02072.x

[B18] Espeland M Murienne J (2011) Diversity dynamics in New Caledonia: towards the end of the museum model? BMC Evolutionary Biology 11: . doi: 10.1186/1471-2148-11-254 10.1186/1471-2148-11-254 PMC3180384 21917169

[B19] Evenhuis N (2008) The insect and spider collections of the world website. http://hbs.bishopmuseum.org/codens/[accessed on November 15, 2013]

[B20] Fauvel A (1904) Faune analytique des Coléoptères de la Nouvelle-Calédonie, 2^e^ partie. Revue d’Entomologie 23: 164–208

[B21] Gargominy O Bouchet P Pascal M Jaffré T Tourneur JC (1996) Conséquences des introductions d’espèces animales et végétales sur la biodiversité en Nouvelle-Calédonie. Revue d’Ecologie (Terre Vie) 51: 375–401

[B22] Gilbert MTP Moore W Melchior L Worobey M (2007) DNA extraction from dry museum beetles without conferring external morphological damage. PLoS ONE 2: . doi: 10.1371/journal.pone.0000272 10.1371/journal.pone.0000272 PMC1803022 17342206

[B23] Grandcolas P Murienne J Robillard T Desutter-Grandcolas L Jourdan H Guilbert E Deharveng L (2008) New Caledonia: a very old Darwinian island? Philosophical Transactions of the Royal Society of London B 363: 3309–3317. doi: 10.1098/rstb.2008.0122 10.1098/rstb.2008.0122 PMC2607381 18765357

[B24] Heads MJ (2008) Panbiogeography of New Caledonia, south-west Pacific: basal angiosperms on basement terranes, ultramafic endemics inherited from volcanic island arcs and old taxa endemic to young islands. Journal of Biogeography 35: 2153–2175. doi: 10.1111/j.1365-2699.2008.01977.x

[B25] Heads MJ (2013) Biogeography of Australasia: A Molecular Analysis. Cambridge University Press. doi: 10.1017/CBO9781139644464

[B26] Hillis DM Bull JJ (1993) An empirical test of bootstrapping as a method for assessing confidence in phylogenetic analysis. Systematic Biology 42: 182–192

[B27] Johanson KA Keijsner M (2008) Phylogeny of the Helicophidae (Trichoptera), with emphasis on the New Caledonian species of *Helicopha*. Systematic Entomology 33: 451–483. doi: 10.1111/j.1365-3113.2008.00423.x

[B28] Jörger KM Schrödl M (2013) How to describe a cryptic species? Practical challenges of molecular taxonomy. Frontiers in Zoology 10: . doi: 10.1186/1742-9994-10-59 10.1186/1742-9994-10-59 PMC4015967 24073641

[B29] Kaszab Z (1982) Die Tenebrioniden Neukaledoniens und der Loyauté-Inseln (Coleoptera). Folia Entomologica Hungarica 28: 1–294

[B30] Kaszab Z (1986) Tenebrioniden (Coleoptera) aus Neukaledonien. Annales Historico-Naturales Musei Nationalis Hungarici 78: 151–175

[B31] Keppel G Lowe AJ Possingham HP (2009) Changing perspectives on the biogeography of the tropical South Pacific: influences of dispersal, vicariance and extinction. Journal of Biogeography 36: 1035–1054. doi: 10.1111/j.1365-2699.2009.02095.x

[B32] Kergoat GJ Delobel A Silvain J-F (2004) Phylogeny and host-specificity of European seed beetles (Coleoptera, Bruchidae), new insights from molecular and ecological data. Molecular Phylogenetics and Evolution 32: 855–865. doi: 10.1016/j.ympev.2004.02.019 15288061 10.1016/j.ympev.2004.02.019

[B33] Kergoat GJ Delobel A Fédière G Le Ru B Silvain J-F (2005) Both host-plant phylogeny and chemistry have shaped the African seed-beetle radiation. Molecular Phylogenetics and Evolution 35: 602–611. doi: 10.1016/j.ympev.2004.12.024 15878129 10.1016/j.ympev.2004.12.024

[B34] Kergoat GJ Le Ru BP Genson G Cruaud C Couloux A Delobel A (2011) Phylogenetics, species boundaries and timing of resource tracking in a highly specialized group of seed beetles (Coleoptera: Chrysomelidae: Bruchinae). Molecular Phylogenetics and Evolution 59: 746–760. doi: 10.1016/j.ympev.2011.03.014 21421066 10.1016/j.ympev.2011.03.014

[B35] Kergoat GJ Soldati L Clamens A-L Jourdan H Jabbour-Zahab R Genson G Bouchard P Condamine FL (2014) Higher level molecular phylogeny of darkling beetle (Coleoptera: Tenebrionidae). Systematic Entomology. doi: 10.1111/syen.12065

[B36] Kuschel G (2008) Curculionoidea (weevils) of New Caledonia and Vanuatu: basal families and some Curculionidae. Zoologica Neocaledonica 6: 99–249

[B37] Ladiges PY Cantrill D (2007) New Caledonia-Australian connections: biogeographic patterns and geology. Australian Systematic Botany 20: 383–389. doi: 10.1071/SB07018

[B38] Lowry PP Munzinger J Bouchet P Géraux J Bauer AM Langrand O Mittermeier RA (2004) New Caledonia. In: Mittermeier RA Robles Gil P Hoffmann M Pilgrim J Brooks T Mittermeier CG Lamoreux JL da Fonseca GAB (Eds) Hotspots revisited: earth’s biologically richest and most threatened ecoregions. CEMEX, Mexico City, Mexico, 193–197

[B39] Malm T Johanson KA (2007) Three new species of *Symphitoneuria* Ulmer (Trichoptera: Leptoceridae) from New Caledonia. In: Proceedings of the XIIth International Symposium of Trichoptera, 181–190

[B40] Malm T Johanson KA (2008a) Revision of the New Caledonian endemic genus *Gracilipsodes* (Trichoptera: Leptoceridae: Grumichellini). Zoological Journal of the Linnean Society 153: 425–452. doi: 10.1111/j.1096-3642.2008.00403.x

[B41] Malm T Johanson KA (2008b) Description of eleven New *Triplectides* species (Trichoptera: Leptoceridae) from New Caledonia. Zootaxa 1816: 1–34

[B42] Matsumoto K Nishikawa N (1986) A revisional study of the species of the genus *Uloma* from Japan, Korea and Taiwan (Tenebrionidae, Coleoptera). Insecta Matsumurana 34: 17–43

[B43] Matthews EG Bouchard P (2008) Tenebrionid beetles of Australia: description of tribes, keys to genera, catalogue of species. Australian Biological Resources Study, Canberra, 398 pp

[B44] Matthews EG Lawrence JF Bouchard P Steiner WE Slipinski SA (2010) 11.14. Tenebrionidae Latreille, 1802. In: Leschen RAB Beutel RG Lawrence JF (Eds) Handbook of Zoology. A Natural History of the Phyla of the Animal Kingdom. Volume IV - Arthropoda: Insecta. Part 38. Coleoptera, Beetles. Volume 2: Systematics (Part 2). Walter de Gruyter, Berlin, 574–659

[B45] McKenna D Sequeira AS Marvaldi AE Farrell BD (2009) Temporal lags and overlap in the diversification of weevils and flowering plants. Proceedings of the National Academy of Sciences of the United States of America 106: 7083–7088. doi: 10.1073/pnas.0810618106 19365072 10.1073/pnas.0810618106 PMC2678426

[B46] Mittermeier RA Werner TB Lees A (1996) New Caledonia – a conservation imperative for an ancient land. Oryx 30: 104–112. doi: 10.1017/S0030605300021487

[B47] Monaghan MT Wild R Elliot M Fujisawa T Balke M Inward DJG Lees DC Ranaivosolo R Eggleton P Barraclough TG Vogler AP (2009) Accelerated species inventory on Madagascar using coalescent-based models of species delineation. Systematic Biology 58: 298–311. doi: 10.1093/sysbio/syp027 20525585 10.1093/sysbio/syp027

[B48] Murienne J Grandcolas P Piulachs MD Bellés X D’Haese C Legendre F Pellens R Guilbert E (2005) Evolution on a shaky piece of Gondwana: is local endemism recent in New Caledonia? Cladistics 21: 2–7. doi: 10.1111/j.1096-0031.2004.00042.x 10.1111/j.1096-0031.2004.00042.x 34892911

[B49] Murienne J Pellens R Budinoff RB Wheeler WC Grandcolas P (2008) Phylogenetic analysis of the endemic New Caledonian cockroach *Lauraesilpha*. Testing competing hypotheses of diversiﬁcation. Cladistics 24: 1–11. doi: 10.1111/j.1096-0031.2008.00204.x

[B50] Myers N Mittermeier RA Mittermeier CG Da Fonseca GAB Kent J (2000) Biodiversity hotspots for conservation priorities. Nature 403: 853–858. doi: 10.1038/35002501 10706275 10.1038/35002501

[B51] Novotny V Drozd P Miller SE Kulfan M Janda M Basset Y Weiblen GD (2006) Why are they so many species of herbivorous insects in tropical rainforests? Science 313: 1115–1118. doi: 10.1126/science.1129237 16840659 10.1126/science.1129237

[B52] Nylander JAA Ronquist F Huelsenbeck JP Nieves-Aldrey JL (2004) Bayesian phylogenetic analysis of combined data. Systematic Biology 53: 47–67. doi: 10.1080/10635150490264699 14965900 10.1080/10635150490264699

[B53] Padial JM Miralles A De la Riva I Vences M (2010) The integrative future of taxonomy. Frontiers in Zoology 7: . doi: 10.1186/1742-9994-7-16 10.1186/1742-9994-7-16 PMC2890416 20500846

[B54] Papadopoulou A Anastasiou I Keskin B Vogler AP (2009) Comparative phylogeography of tenebrionid beetles in the Aegean archipelago: the effect of dispersal ability and habitat preference. Molecular Ecology 18: 2503–2517. doi: 10.1111/j.1365-294X.2009.04207.x 19457194 10.1111/j.1365-294X.2009.04207.x

[B55] Papadopoulou A Anastasiou I Vogler AP (2010) Revisiting the insect mitochondrial molecular clock: the mid-Aegean trench calibration. Molecular Biology and Evolution 27: 1659–1672. doi: 10.1093/molbev/msq051 20167609 10.1093/molbev/msq051

[B56] Pascal M Richer de Forges B Le Guyader H Simberloff D (2008) Mining and other threats to the New Caledonia biodiversity hotspot. Conservation Biology 22: 498–499. doi: 10.1111/j.1523-1739.2008.00889.x 18402591 10.1111/j.1523-1739.2008.00889.x

[B57] Pelletier B (2006) Geology of the New Caledonia region and its implications for the study of the New Caledonian biodiversity. Documents Scientifiques et Techniques de l’IRD II 7: 17–30

[B58] Pillon Y Munzinger J Amir H Lebrun M (2010) Ultramaﬁc soils and species sorting in the ﬂora of New Caledonia. Journal of Ecology 98: 1108–1116. doi: 10.1111/j.1365-2745.2010.01689.x

[B59] Pillon Y (2012) Time and tempo of diversiﬁcation in the ﬂora of New Caledonia. Botanical Journal of the Linnean Society 170: 288–298. doi: 10.1111/j.1095-8339.2012.01274.x

[B60] Pons J Barroclough TG Gomez-Zurita J Cardoso A Duran DP Hazell S Kamoun S Sumlin WD Vogler AP (2006) Sequence-based species delimitation for the DNA taxonomy of undescribed insects. Systematic Biology 55: 595–609. doi: 10.1080/10635150600852011 16967577 10.1080/10635150600852011

[B61] Oláh J Johanson KA (2008) Generic review of Hydropsychinae, with description of *Schmidopsyche*, new genus, 3 new genus clusters, 8 new species groups, 4 new species clades, 12 new species clusters and 62 new species from the Oriental and Afrotropical regions (Trichoptera: Hydropsychidae). Zootaxa 1802: 1–248

[B62] Robillard T Desutter-Grandcolas L (2006) Phylogeny of the cricket subfamily Eneopterinae (Orthoptera, Grylloidea, Eneopteridae) based on four molecular loci and morphology. Molecular Phylogenetics and Evolution 40: 643–661. doi: 10.1016/j.ympev.2005.10.019 16713307 10.1016/j.ympev.2005.10.019

[B63] Robillard T Desutter-Grandcolas L (2008) Systematics of *Matuanus* Gorochov (Grylloidea, Podoscirtidae, Podoscirtinae) from New Caledonia: new data and the analysis of venation diversity. In: Grandcolas P (Ed) Zoologia Neocaledonica 6, Systematics and Biodiversity in New Caledonia.Mémoires du Muséum National d’Histoire Naturelle 196: 273–289

[B64] Schellart WP Lister GS Toy VG (2006) A Late Cretaceous and Cenozoic reconstruction of the Southwest Pacific region: Tectonics controlled by subduction and slab rollback processes. Earth-Science Reviews 76: 191–233. doi: 10.1016/j.earscirev.2006.01.002

[B65] Schlick-Steiner BC Steiner FM Seifert B Stauffer C Christian E Crozier RH (2010) Integrative taxonomy: a multisource approach to exploring biodiversity. Annual Review of Entomology 55: 421–438. doi: 10.1146/annurev-ento-112408-085432 10.1146/annurev-ento-112408-085432 19737081

[B66] Sharma P Giribet G (2009) A relict in New Caledonia: Phylogenetic relationships of the family Troglosironidae (Opiliones: Cyphophthalmi). Cladistics 25: 279–294. doi: 10.1111/j.1096-0031.2009.00252.x 10.1111/j.1096-0031.2009.00252.x 34879611

[B67] Smith SA Sadlier RA Bauer AM Austin CC Jakman T (2007) Molecular phylogeny of the scincid lizards of New Caledonia and adjacent areas: Evidence for a single origin of the endemic skinks of Tasmantis. Molecular Phylogenetics and Evolution 43: 1151–1166. doi: 10.1016/j.ympev.2007.02.007 17400482 10.1016/j.ympev.2007.02.007

[B68] Stork N Habel JC (2014) Do biodiversity hotspots protect more than tropical forest plants and vertebrates? Journal of Biogeography 41: 421–428. doi: 10.1111/jbi.12223

[B69] Stamatakis A (2006) RAxML-VI-HPC: maximum likelihood-based phylogenetic analyses with thousands of taxa and mixed models. Bioinformatics 22: 2688–2690. doi: 10.1093/bioinformatics/btl446 16928733 10.1093/bioinformatics/btl446

[B70] Silvestro D Michalak I (2012) raxmlGUI: a graphical front-end for RaxML. Organisms Diversity and Evolution 12: 335–337. doi: 10.1007/s13127-011-0056-0

[B71] Swenson U Backlund A McLoughghlin S Hill RS (2001) *Nothofagus* biogeography revisited with special emphasis on the enigmatic distribution of subgenus *Brassospora* in New Caledonia. Cladistics 17: 28–47. doi: 10.1111/j.1096-0031.2001.tb00109.x

[B72] Vieites DR Wollenberg KC Andreone F Köhler J Glaw F Vences M (2009) Vast underestimation of Madagascar’s biodiversity evidenced by an integrative amphibian inventory. Proceedings of the National Academy of Science of the United States of America 106: 8267–8272. doi: 10.1073/pnas.0810821106 10.1073/pnas.0810821106 PMC2688882 19416818

[B73] Wild AL Maddison DR (2008) Evaluating nuclear protein-coding genes for phylogenetic utility in beetles. Molecular Phylogenetics and Evolution 48: 877–891. doi: 10.1016/j.ympev.2008.05.023 18644735 10.1016/j.ympev.2008.05.023

[B74] Zhang J Kapli P Pavlidis P Stamatakis A (2013) A general species delimitation method with applications to phylogenetic placements. Bioinformatics 29: 2869–2876. doi: 10.1093/bioinformatics/btt499 23990417 10.1093/bioinformatics/btt499 PMC3810850

